# Seven species new to science and one newly recorded species of the ant genus *Myrmica* Latreille, 1804 from China, with proposal of a new synonym (Hymenoptera, Formicidae)

**DOI:** 10.3897/zookeys.551.6005

**Published:** 2016-01-11

**Authors:** Zhilin Chen, Shanyi Zhou, Jianhua Huang

**Affiliations:** 1 Guangxi Key Laboratory of Rare and Endangered Animal Ecology, Guangxi Normal University, Guilin, 541004, China; 2 Key Laboratory of Ecology of Rare and Endangered Species and Environmental Protection (Guangxi Normal University), Ministry of Education, Guilin, 541004, China; 3 College of Life Sciences, Guangxi Normal University, Guilin 541004, China; 4 Key Laboratory of Cultivation and Protection for Non-Wood Forest Trees (Central South University of Forestry and Technology), Ministry of Education, Changsha, Hunan 410004, China; 5 College of Forestry, Central South University of Forestry and Technology, Changsha, Hunan 410004, China

**Keywords:** Formicidae, Myrmica, new species, new synonym, new Chinese record

## Abstract

Seven new species of the genus *Myrmica* Latreille, 1804 are described from China: *Myrmica dongi* **sp. n.**, *Myrmica huaii* **sp. n.**, *Myrmica liui* **sp. n.**, *Myrmica mifui* **sp. n.**, *Myrmica oui* **sp. n.**, *Myrmica wangi* **sp. n.** and *Myrmica yani* **sp. n**. *Myrmica forcipata* Karawaiew, 1931 is recorded from China for the first time, while *Myrmica zhengi* Ma & Xu, 2011 is synonymized with *Myrmica luteola* Kupyanskaya, 1990. Identification keys based on worker caste are provided to the *Myrmica* species of China and the *pachei*-group species of the Old World, respectively.

## Introduction

*Myrmica* Latreille, 1804 is a large genus belonging to the family Formicidae, with 200 species and 12 subspecies known worldwide to date (Bolton 2014). Although there was confusion of the concept of the genus *Myrmica* before the start of the twentieth century (Nylander 1846a, 1846b, 1849, 1856, 1857, Curtis 1854), which led to the additions to the genus of numerous taxa that did not truly belong to *Myrmica* (Radchenko and Elmes 2010), the works by Finzi (1926), Santschi (1931) and Arnol'di (1934) clarified the genus definition. The first revision of the genus *Myrmica* was provided by Weber (1947, 1948, 1950), who paid most of his attention to the Nearctic species but presented a comprehensive synopsis of the Palearctic species as well. From then on, a series of revisions were devoted to the genus *Myrmica* in the subsequent decades (Sadil 1952, Yarrow 1955, Arnol'di 1970, 1976, Kutter 1970, 1973, Francoeur 1981, 2007, Kupyanskaya 1986a, 1986b, Bolton 1988, Seifert 1988, 2003, 2011, Czekes et al. 2013). Besides the efforts described above, a more extensive revisionary project was launched by Redchenko and his colleagues. Radchenko's interest was first focused on the *Myrmica* species of the central and eastern Palaearctic region (Radchenko 1994a−f, Radchenko et al. 1997), but soon extended to the Himalaya, south-east Asia and the whole Oriental region (Radchenko and Elmes 1998, 1999a, 1999b, Elmes and Radchenko 1998). With the cardinal revisions of more species groups (Radchenko and Elmes 2001a, 2003a, 2004, 2009a, 2009b, Radchenko et al. 2002, Radchenko et al. 2006) and regional faunistic investigations (Radchenko and Elmes 2001, 2002, 2003, 2009c, Radchenko et al. 2001, Elmes et al. 2001, Elmes et al. 2002, Radchenko et al. 2006, Radchenko et al. 2008a, 2008b, Elmes and Radchenko 2009), many new species were described from the regions with poorly known *Myrmica* fauna (e.g. China, Korea, Vietnam, Turkey, Sicily etc.), and the taxonomy of *Myrmica* in the Old World culminated with the publication of the monograph by Radchenko and Elmes (2010), in which a total of 147 species were recognized, including five fossil species from the European late Eocene ambers. Stappen (2014) made a systemic evaluation on work of Radchenko and Elmes (2010) with some modifications, changes. On the basis of the work of Radchenko and Elmes (2010), further research was conducted in the Himalayan region (Bharti 2012a, 2012b, Bharti and Sharma 2011a, 2011b, 2011c, 2013), resulting in the discovery of nine new species in total.

The first Chinese species of the genus *Myrmica*, i.e. *Myrmica tibetana* Mayr, 1889, was described from Xizang. Ruzsky (1915) described eight species/subspecies and added *Myrmica smythiesii* Forel, 1902 to the Xizang fauna. Further work was carried out on the *Myrmica* fauna of China by later authors (Viehmeyer 1922, Wheeler 1928, 1929, 1930a, Donisthorpe 1929, Santschi 1937, Eidmann 1941, Wu and Wang 1995, Tang et al. 1995, Xia and Zheng 1997, Elmes and Radchenko 1998, Wei et al. 1999, Collingwood and Heatwole 2000, Wei et al. 2001b, Chang and He 2001b, Zhou 2001, Zhou and Huang 2002, Xu 2002, Radchenko et al. 2001, Huang et al. 2004, Li et al. 2005, Tie and Xu 2004, Tie and Xu 2005, Wang et al. 2005, Zhou 2005, Zhou 2006, Radchenko et al. 2008, Wang et al. 2009, Radchenko and Elmes 2009a, Radchenko and Elmes 2010, Zhou and Qian 2010, Xu et al. 2011, Zhang et al. 2011, Zhou 2013), and the sporadic results were summarized in several checklists (Wheeler 1930b, Wu 1941, Chou and Terayama 1991, Huang and Zhou 2007, Guenard and Dunn 2012). Meanwhile, nomenclatural changes were made in various revisionary works. In detail, *Myrmica kozlovi mekongi*, *Myrmica kozlovi subbrevispinosa* and *Myrmica kozlovi subalpina* were synonymized with *Myrmica kozlovi*, *Myrmica taediosa* with *Myrmica transsibirica*, *Myrmica tibetana furva* with *Myrmica tibetana*, *Myrmica chinensis* and *Myrmica helleri* with *Myrmica kurokii* (Radchenko and Elmes 2010), *Myrmica everesti* with *Myrmica rupestris*, *Myrmica specularis* with *Myrmica kozlovi* (Radchenko and Elmes, 2001), *Myrmica kurokii tipuna* with *Myrmica arisana* (Elmes and Radchenko 1998), *Myrmica sinica* with *Myrmica excelsa* (Radchenko et al. 2008), *Myrmica limanica* with *Myrmica gallienii* (Collingwood 1979), *Myrmica smythiesii exigua* Ruzsky, 1915 was replaced with *Myrmica ruzskyana* (Radchenko and Elmes 2010); the following four taxa were raised to species: *Myrmica smythiesii bactriana*, *Myrmica margaritae serica*, *Myrmica margaritae pulchella* (Radchenko and Elmes 2010), *Myrmica rugosa arisana* (Elmes & Radchenko, 1998); *Myrmica rubra khamensis* (= *Myrmica ruginodis khamensis*) was considered as *incertae sedis* in *Myrmica* (Radchenko and Elmes 2010); *Myrmica margaritae inornata* Menozzi, 1941 was determined as *nomen nudum* (Bolton 1995); the records for China of *Myrmica gallienii* (Wei et al. 2001b, Chang and He 2001b), *Myrmica inezae* (Wei et al. 1999, Wei et al. 2001b), *Myrmica jessensis* (Wu and Wang 1995, Wei et al. 2001b), *Myrmica lobicornis* (Eidmann 1941, Wei et al. 2001b), *Myrmica margaritae* (Eidmann 1941, Wu and Wang 1995, Zhou 2001, Wei et al. 2001b), *Myrmica smythiesi cachmiriensis* (Eidmann 1941) and *Myrmica wesmaeli* (Chang and He 2001b) were deemed misidentifications, so they were excluded from Chinese fauna (Radchenko and Elmes 2010). In terms of all taxonomic decisions above-mentioned, 46 *Myrmica* species are recognized from China so far and *Myrmica ruginodis khamensis* was considered an unidentifiable taxon recently (Radchenko and Elmes 2010). However, there are at least 104 species found in the surrounding regions of China which may be recorded in China in the near future, indicating that the diversity of *Myrmica* in China is extremely high. The *Myrmica* fauna of China is still poorly known, and many more species certainly remain to be found.

In this paper, seven new and one newly recorded *Myrmica* species are described from China. *Myrmica zhengi* Ma & Xu, 2011 is considered as a junior synonym of *Myrmica luteola* Kupyanskaya, 1990, leading to an increase of the known Chinese *Myrmica* species to 54.

## Materials and methods

This study is based on the specimens deposited in the Insect Collection of Guangxi Normal University, Guilin, China. Digital images of the specimens were taken with a Nikon AZ100 microscope. All measurements are in millimeters. Standard measurements and indices are mostly defined by Radchenko and Elmes (2010):

HL
 length of the head in full face view, measured in a straight line from the middle of anterior clypeal margin to the middle of posterior margin.

HW
 maximum width of the head in full face view behind the eyes.

FW
 minimum distance of frons between the frontal carinae.

FLW
 maximum distance between the outer borders of the frontal lobes.

SL
 maximum straight length of the antennal scape in profile view.

PW
 maximum width of pronotum in dorsal view.

ML
 length of mesosoma in profile, measured from the point at which the pronotum meets the cervical shield to the posterior basal angle of the metapleuron.

PL
 maximum length of petiole in dorsal view.

PH
 maximum height of petiole in profile view.

ESL
 straight length of propodeal spine in profile view, from its tip to the deepest point of the propodeal constriction at the base of the spine.

CI
 HL/HW


ESLI
 ESL/HW


FI
 FW/HW


FLI
 FLW/FW


SI1
 SL/HL


SI2
 SL/HW


## Taxonomic checklist of *Myrmica* species in China

A list of species of *Myrmica* ants currently known from China is presented, according to the literatures and our collections. For each species the distributed places of China and the citations are mentioned. The list is arranged alphabetically.

***Myrmica angulata* Radchenko, Zhou & Elmes, 2001**

**Distribution.** Guangxi (Radchenko et al. 2001, Chen and Zhou 2007) and Hubei (Lyu and Cho 2003).

***Myrmica angulinodis* Ruzsky, 1905**

**Distribution.** Gansu (Chang and He 2001a), Inner Mongolia (Collingwood and Heatwole 2002), Qinghai (Tie and Xu 2004), Xinjiang (Collingwood and Heatwole 2002).

***Myrmica arisana* Wheeler, 1930**

**Distribution.** Taiwan (Wheeler 1930, Huang and Zhou 2007).

***Myrmica bactriana* Ruzsky, 1915**

**Distribution.** Qinghai (Radchenko and Elmes 2010), Xinjiang (Huang and Zhou 2007), Xizang (Xu et al. 2011, Zhang et al. 2011).

***Myrmica curiosa* Radchenko, Zhou & Elmes, 2008**

**Distribution.** Hunan (Radchenko et al. 2008), Sichuan (Radchenko et al. 2008) and Yunnan (Radchenko et al. 2008).

***Myrmica deplanata* Emery, 1921**

**Distribution.** Ningxia (Tie and Xu 2004) and Qinghai (Chang and He 2001b, Huang and Zhou 2007).

***Myrmica dongi* sp. n.**

**Distribution.** Xizang.

***Myrmica draco* Radchenko, Zhou & Elmes, 2001**

**Distribution.** Guangdong (Huang and Zhou 2007), Guangxi (Radchenko et al. 2001), Henan (Li et al. 2005), Shaanxi (Radchenko and Elmes 2010) and Yunnan (Radchenko and Elmes 2010).

***Myrmica eidmanni* Menozzi, 1930**

**Distribution.** Liaoning (Radchenko and Elmes 2010), Heilongjiang (Radchenko and Elmes 2010), Jilin (Radchenko and Elmes 2010).

***Myrmica excelsa* Kupyanskaya, 1990**

= *Myrmica sinica* Wu & Wang, 1995

**Distribution.** Gansu (Chen 2008), Henan (Huang and Zhou 2007), Hubei (Wang and Zhao 2009), Shaanxi (Tie and Xu 2004, Wei et al. 2001a), Shandong (Wei et al. 2001b).

***Myrmica forcipata* Karavajev, 1931 (new record for China)**

**Distribution.** Ningxia.

***Myrmica heterorhytida* Radchenko & Elmes, 2009**

**Distribution.** Yunnan (Radchenko and Elmes 2010).

***Myrmica hlavaci* Radchenko & Elmes, 2009**

**Distribution.** Sichuan (Radchenko and Elmes 2010).

***Myrmica huaii* sp. n.**

**Distribution.** Shaanxi.

***Myrmica koreana* Elmes, Radchenko & Kim, 2001**

**Distribution.** North east part of China (Radchenko and Elmes 2010).

***Myrmica kotokui* Forel, 1911**

**Distribution.** North east part of China (Radchenko and Elmes 2010).

***Myrmica kozlovi* Ruzsky, 1915**

= *Myrmica kozlovi mekongi* Ruzsky, 1915

= *Myrmica kozlovi subalpina* Ruzsky, 1915

= *Myrmica kozlovi subbrevispinosa* Ruzsky, 1915

**Distribution.** Xizang (Huang and Zhou 2007).

***Myrmica kurokii* Forel, 1907**

= *Myrmica chinensis* Viehmeyer, 1922

= *Myrmica helleri* Viehmeyer, 1922

**Distribution.** Sichuan (Huang and Zhou 2007, Radchenko and Elmes 2010).

***Myrmica liui* sp. n.**

**Distribution.** Inner Mongolia.

***Myrmica luteola* Kupyanskaya, 1990**

= *Myrmica zhengi* Ma & Xu, 2011, **syn. n.**

**Distribution.** Shaanxi (Ma and Xu 2011).

***Myrmica mifui* sp. n.**

**Distribution.** Shaanxi.

***Myrmica mirabilis* Elmes & Radchenko, 1998**

**Distribution.** Taiwan (Huang and Zhou 2007).

***Myrmica mixta* Radchenko & Elmes, 2008**

**Distribution.** Sichuan (Radchenko et al. 2008).

***Myrmica multiplex* Radchenko & Elmes, 2009**

**Distribution.** Shaanxi (Radchenko and Elmes 2010).

***Myrmica oui* sp. n.**

**Distribution.** Guizhou.

***Myrmica pararitae* Radchenko & Elmes, 2008**

**Distribution.** Sichuan (Radchenko et al. 2008).

***Myrmica phalacra* Radchenko & Elmes, 2009**

**Distribution.** Shaanxi (Radchenko and Elmes 2010).

***Myrmica pleiorhytida* Radchenko & Elmes, 2009**

**Distribution.** Yunnan (Radchenko and Elmes 2010).

***Myrmica poldii* Radchenko & Rigato, 2008**

**Distribution.** Sichuan (Radchenko et al. 2008).

***Myrmica polyglypta* Radchenko & Rigato, 2008**

**Distribution.** Yunnan (Radchenko et al. 2008).

***Myrmica pulchella* Santschi, 1937**

= *Myrmica formosae* Wheeler W.M., 1929

**Distribution.** Taiwan (Hua 2006, Huang and Zhou 2007).

***Myrmica ritae* Emery, 1889**

**Distribution.** Sichuan (Radchenko et al. 2008).

***Myrmica rubra* (Linnaeus, 1758)**

**Distribution.** Gansu (Chen 2008), Ningxia (Tie and Xu 2004, Xin et al. 2011), Qinghai (Chang and He 2001b), Shaanxi (Tie and Xu 2004), Shaanxi (Wei et al. 2001b), Xinjiang (Wu et al. 2004), Xizang (Xu et al. 2011, Zhang et al. 2011).

***Myrmica ruginodis* Nylander, 1846**

**Distribution.** Gansu (Collingwood 1962), Heilongjiang (Wei et al. 2001b; Yasumatsu 1941), Henan (Li et al. 2005), Hunan (Huang and Zhou 2007, Huang et al. 2005), Jilin (Wei et al. 2001b), Ningxia (Ma et al. 2008, Xin et al. 2011), Shaanxi (Liu et al. 1999).

***Myrmica ruzskyana* Radchenko & Elmes, 2010**

**Distribution.** Xinjiang (Huang and Zhou 2007, Radchenko and Elmes 2010).

***Myrmica saposhnikovi* Ruzsky, 1904**

**Distribution.** Xizang (Huang and Zhou 2007).

***Myrmica scabrinodis* Nylander, 1846**

**Distribution.** Xinjiang (Xia and Zheng 1997).

***Myrmica schencki* Viereck, 1903**

**Distribution.** Sichuan (Collingwood 1962), Xinjiang (Wu et al. 2004).

***Myrmica schulzi* Radchenko & Elmes, 2009**

**Distribution.** Shaanxi (Radchenko and Elmes 2010).

***Myrmica sculptiventris* Radchenko & Elmes, 2009**

**Distribution.** Sichuan (Radchenko and Elmes 2010).

***Myrmica serica* Wheeler, 1928**

**Distribution.** Shaanxi (Radchenko et al. 2006), Shaanxi (Radchenko et al. 2001), Yunnan (Radchenko et al. 2001) and Taiwan (Huang and Zhou 2007, Radchenko et al. 2001).

***Myrmica sinensis* Radchenko, Zhou & Elmes, 2001**

**Distribution.** Guangxi (Radchenko et al. 2001, Chen and Zhou 2007) and Henan (Li et al. 2005).

***Myrmica sinoschencki* Radchenko & Elmes, 2008**

**Distribution.** Sichuan (Radchenko et al. 2008).

***Myrmica stangeana* Ruzsky, 1902**

**Distribution.** Xinjiang (Xia and Zheng 1997).

***Myrmica sulcinodis* Nylander, 1846**

**Distribution.** Gansu (Chang and He 2001b), Inner Mongolia (Wei et al. 2001b), Ningxia (Tie and Xu 2004, Xin et al. 2011), Qinghai (Tie and Xu 2004).

***Myrmica taibaiensis* Wei, Zhou & Liu, 2001**

**Distribution.** Shaanxi (Wei et al. 2001b).

***Myrmica tibetana* Mayr, 1889**

**Distribution.** Xizang (Huang and Zhou 2007).

***Myrmica transsibirica* Radchenko, 1994**

**Distribution.** Heilongjiang (Radchenko and Elmes 2010), Jilin (Radchenko and Elmes 2010).

***Myrmica urbanii* Radchenko & Elmes, 1998**

**Distribution.** Hubei (Wang et al. 2005).

***Myrmica vandeli* Bondroit, 1920**

**Distribution.** Xinjiang (Xia and Zheng 1997).

***Myrmica wangi* sp. n.**

**Distribution.** Shaanxi.

***Myrmica weii* Radchenko & Zhou, 2008**

**Distribution.** Shaanxi (Radchenko et al. 2008).

***Myrmica yani* sp. n.**

**Distribution.** Guizhou.

***Myrmica yunnanensis* Radchenko & Elmes, 2009**

**Distribution.** Yunnan (Radchenko and Elmes 2010).

**Unidentifiable names and *incertae sedis***

**Myrmica ruginodis var. khamensis Ruzsky, 1915**

**Distribution.** China: Xizang (Huang and Zhou 2007).

**Doubtful species whose presence in China could not be verified.**

***Myrmica aloba* Forel, 1909**

**Distribution.** Xizang (Tie and Xu 2004).

***Myrmica inezae* Forel, 1902**

**Distribution.** Shaanxi (Tie and Xu 2004), Sichuan (Alonso et al. 2009), Yunnan (Radchenko 2004).

***Myrmica gallienii* (Wei et al. 2001b; Chang and He 2001b)**

**Distribution.** Gansu (Chang and He 2001b), Ningxia (Chang and He 2001b, Tie and Xu 2004), Shaanxi (Tie and Xu 2004) and Xinjiang (Wu et al. 2004).

***Myrmica jessensis* Forel, 1901**

**Distribution.** Gansu (Chen 2008), Hebei (Wei et al. 2001b), Heilongjiang (Wei et al. 2001b), Hubei (Hua 2006), Hunan (Huang and Zhou 2007, Huang et al. 2005), Inner Mongolia (Wei et al. 2001b), Jilin (Wei et al. 2001b), Ningxia (Wang 2009), Shaanxi (Tie and Xu 2004), Sichuan (Wei et al. 2001b), Xizang (Xu et al. 2011, Zhang et al. 2011).

***Myrmica lobicornis* Nylander, 1846**

**Distribution.** Beijing (Wei et al. 2001b), Gansu (Chen 2008), Hebei (Hua 2006), Heilongjiang (Wei et al. 2001b), Henan (Li et al. 2005), Inner Mongolia (Wei et al. 2001b), Jilin (Wei et al. 2001b), Liaoning (Wei et al. 2001b), Ningxia (Tie and Xu 2004, Xin et al. 2011), Qinghai (Zhang and Zheng 2002), Shaanxi (Tie and Xu 2004), Shaanxi (Wei et al. 2001b), Sichuan (Zhang and Zheng 2002).

***Myrmica margaritae* Emery, 1889**

**Distribution.** Anhui (Wei et al. 2001b), Guangxi (Huang and Zhou 2007), Hebei (Wei et al. 2001b), Henan (Li et al. 2005), Hubei (Huang and Zhou 2007), Hunan (Wei et al. 2001, Huang et al. 2005), Shaanxi (Tie and Xu 2004), Sichuang (Zhang et al. 2011, Liu et al. 2011), Yunnan (Yang et al. 2004, Xu 2002), Zhejiang (Wei et al. 2001b) and Taiwan (Huang and Zhou 2007).

***Myrmica rugosa* Mayr, 1855**

**Distribution.** China: Fujian (Huang and Zhou 2007), Xizang (Huang and Zhou 2007), Taiwan (Huang and Zhou 2007).

***Myrmica rupestris* Forel, 1902**

**Distribution.** Xizang (Donisthorpe 1929).

***Myrmica smythiesii* Forel, 1902**

**Distribution.** Xizang (Xu et al. 2011, Zhang et al. 2011).

***Myrmica tulinae* Elmes, Radchenko & Aktaç, 2002**

**Distribution.** Shaanxi (Tie and Xu 2005).

***Myrmica wesmaeli* Bondroit, 1918**

**Distribution.** Ningxia (Chang and He 2001b, Tie and Xu 2004) and Qinghai (Chang and He 2001b, Huang and Zhou 2007).

## Taxonomy

### Myrmica luteola

Taxon classificationAnimaliaHymenopteraFormicidae

Kupyanskaya, 1990

Figures 1–4

Myrmica luteola Kupyanskaya, 1990: 103, Figs 16, 17 (w.q.) RUSSIA; 2003: 239; Radchenko and Elmes 2010: 197.
Myrmica zhengi Ma & Xu, 2011: 795, figs 1−5 (w.m.) CHINA. **syn. n.**

#### Material examined.

***Myrmica zhengi***: paratypes, 4 workers: Foping Nature Reserve (33°42'N, 107°48'E), Shaanxi Province, China. 23.vii.2006, leg. Libin Ma, No. G060078; 3 workers: Qin Ling, Shaanxi Province, China. 27.vii.2006, leg. Zhao Tan, No. G060158; 1 worker, identification and presentation by Alexander G. Radchenko, but lack of collecting information.

#### Differential diagnosis.

As Radchenko and Elmes (2010) noted, this species is very easy to distinguish from all other *Myrmica* species due to its unique features, i.e. strongly reduced and simple non-pectinate spurs on the middle and hind tibiae, and somewhat developed ventral petiolar and postpetiolar processes. Moreover, the workers show another feature that very rarely occurs in *Myrmica* species: the base of the first gastral tergite is distinctly longitudinally striated. Ma and Xu (2011) described *Myrmica zhengi* from Shaanxi perhaps without reading the papers by Kupyanskaya (1990) and Radchenko et al. (2003a, 2010). These three important references are also not cited by Ma & Xu, so that they missed the key features. After a careful comparison of the five workers paratype and one queen paratype of *Myrmica zhengi* with the original morphological descriptions and the identified specimens of *Myrmica luteola* by Prof. Alexander G. Radchenko (Museum and Institute of Zoology Polish Academy of Sciences, Poland), we found no differences between them; therefore, we propose *Myrmica zhengi* as a junior synonym of *Myrmica luteola*.

**Figures 1–4.**
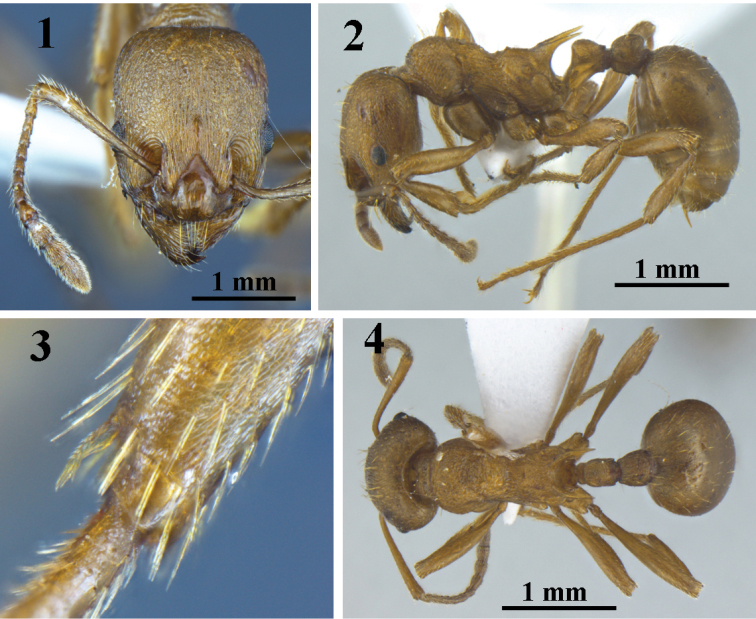
*Myrmica zhengi* Ma & Xu, 2011 = *Myrmica luteola* Kupyanskaya, 1990. worker (paratype) (G060078). **1** head in full-face view **2** body in profile view **3** spurs of hind tibiae **4** body in dorsal view.

### Myrmica forcipata

Taxon classificationAnimaliaHymenopteraFormicidae

Karavajev, 1931

Figures 5–7

Myrmica forcipata Karavajev, 1931: 105, fig. 2 (w.) RUSSIA; Radchenko and Elmes 2010: 134.

#### Material examined.

5 workers, Xiaowutai Mountain, Hebei province, China, 39°00'25"N, 113°35'46"E, 1751m, 21.vi.2009, leg. Shanyi Zhou, No. G090211.

#### Differential diagnosis.

This species is similar to *Myrmica angulinodis*, but differs from the latter by the distinct, though not large, vertical lobe at the scape bend. This species was previously known only from south and east Siberia, Mongolia, but absent in the Russian far east. Herein this species is recorded from China for the first time.

**Figures 5–7.**
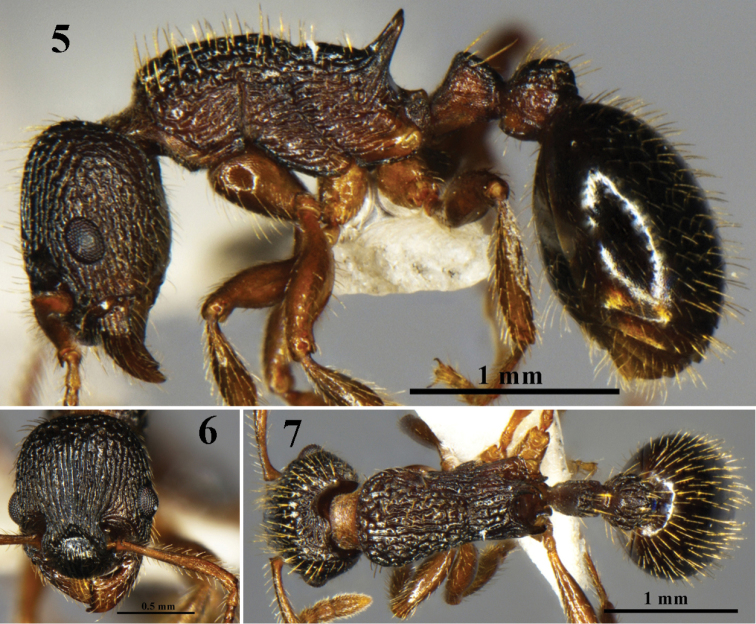
*Myrmica forcipata* Karavajev, 1931. Worker (No. G090211). **5** body in profile view **6** head in full-face view **7** body in dorsal view.

### Myrmica dongi sp. n.

Taxon classificationAnimaliaHymenopteraFormicidae

http://zoobank.org/6B0F6901-D0FD-4CF4-8D7A-0DC06960C419

Figures 8–13

#### Type material.

**Holotype worker.** Sejila Mountain, Linzhi County, Xizang Autonomous Region, 29°40'00"N, 94°23'08"E, 4200m, 14.vi.2009, leg. Shuang Zhao, No. G090156. **Paratypes.** 1 worker, 11.vi.2009, No. G090156; 1 worker, 14.vi.2009, No. G090137; 1 worker, 15.vi.2009, No. G090141; 1 queen, 17.vi.2009, No. G090149; the locality and collector the same as holotype.

#### Measurements and descriptions.

**Figures 8–13.**
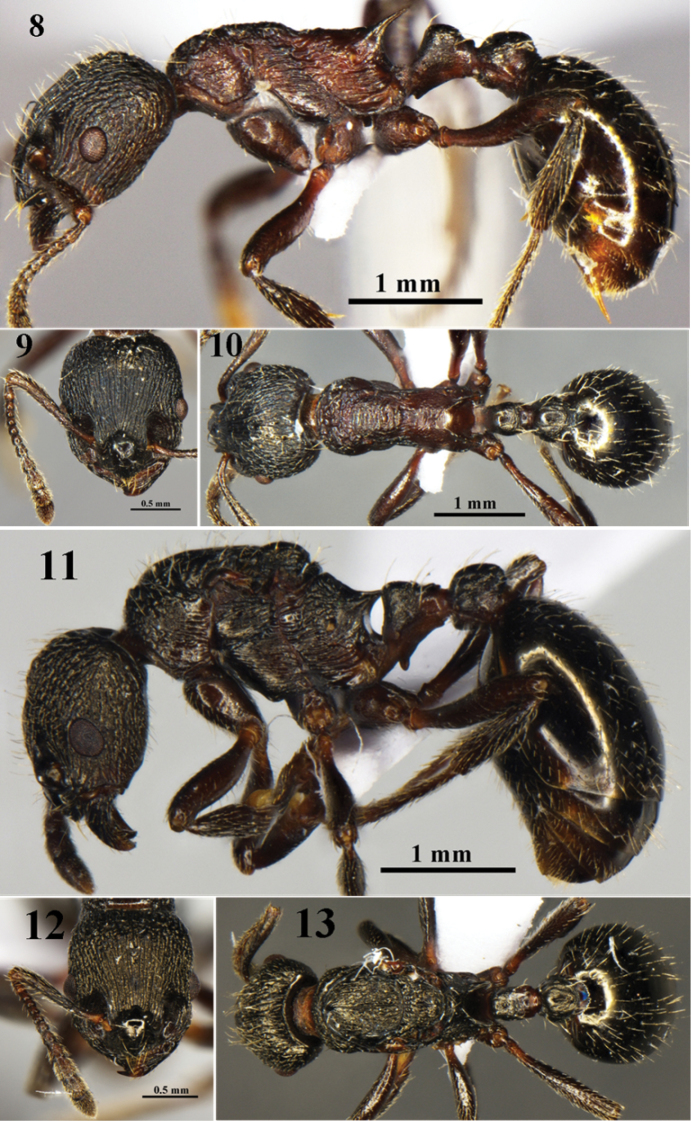
*Myrmica dongi* sp. n. **8−10**: worker (No. G090156); **11−13**: queen (No. G090149). **8, 11** body in profile view **9, 12** head in full−face view **10, 13** body in dorsal view.

**Holotype worker** (Figs 8–10). HL 1.40, HW 1.25, FW 0.50, FLW 0.53, SL 1.15, PW 0.87, ML 1.75, PL 0.50, PH 0.45, ESL 0.40, CI 1.12, FI 0.40, FLI 1.06, SI_1_ 0.82, SI_2_ 0.92, ESLI 0.32. **Paratype workers** (n = 3). HL 1.30−1.41, HW 1.10−1.25, FW 0.48−0.50 FLW 0.50−0.53, SL 1.10−1.12, PW 0.82−0.90, ML 1.70−1.79, PL 0.42−0.51, PH 0.42−0.58, ESL 0.37−0.45, CI 1.13−1.17, FI 0.35−0.36, FLI 1.04−1.06, SI_1_ 0.80−0.87, SI_2_ 0.89−0.94, ESLI 0.27−0.34.

**Paratype queen** (Figs 11–13). HL1.25, HW 1.18, FW 0.53, FLW 0.55, SL 1.17, PW 0.95, ML 1.88, PL 0.5, PH 0.20, ESL 0.20, CI 1.06, FI 0.45, FLI 1.04, SI_1_ 0.93, SI_2_ 0.99, ESLI 0.17.

**Holotype worker.** Head longer than broad, with very weakly convex sides, almost straight posterior margin and rounded posterior corners; anterior clypeal margin rounded, slightly prominent, not notched medially. Frontal carinae curved outwards to merge with the rugae that surround antennal sockets. Frons wide, frontal lobes not extended. Antennal scape relatively long (SI_2_ = 0.92), slightly shorter than head width, gradually though distinctly curved at the base, without any trace of lobe or carina.

Mesosoma robust, promesonotum in profile view slightly convex, promesonotal suture in dorsal view indistinct. Metanotal groove distinct, wide, but shallow. Propodeal lobes rounded. Propodeal spines relatively short, straight, sharp, directly backwards at an angle of less than 45º. Petiole high, with very short peduncle; petiolar node in profile view cylindric, anterior surface concave, dorsum of node slightly convex, with a distinct broad dorsal plate, posterior surface steep. Postpetiole subglobular, with anterior and dorsal surfaces forming a regular arch. Spurs of middle and hind tibiae well-developed and pectinate. Frons with dense, fine, slightly sinuous, longitudinal rugae, number of rugae between frontal carinae level with the eyes *ca.* 20, posterior part of the head and its sides with fine reticulation, spaces between rugae sparsely superficially punctate, appearing more or less shiny and never dull. Clypeus with longitudinal rugae, spaces between them shiny. Frontal triangle smooth and shiny. Pronotal dorsum with reticulation, lateral sides longitudinally rugose-punctate. Mesonotal and propodeal dorsum with < 20 moderately coarse transverse sinuous rugae. Lower parts of mesopleura and lateral sides of propedeum with longitudinal rugae. Spaces between rugae on mesosoma with fine punctures, but appearing quite shiny. Petiole and postpetiole dull, densely punctate.

Head without subdecumbent pilosity at lateral margins, posterior margin with erect to suberect long hairs, genae with a few long hairs; dorsum of mesosoma with long hairs; petiole with 5−6 long hairs and a few short hairs. Antennal scapes and tibiae with subdecumbent hairs. Body colored blackish-brown, appendages somewhat lighter.

**Paratype workers.** With similar morphological characters as holotype, but in some individuals, color reddish-brown to yellowish-brown; petiole only with 3 long hairs.

**Paratype queen.** Queen generally similar to workers in the shape and sculpture of the head, frontal lobes, propodeal spines (which are more blunt at the apex), petiole and postpetiole. Mesosoma long and low, coarsely sculptured; anterior half of scutum with sinuous longitudinal rugae and reticulations; posterior half of scutum, scutellum and propodeal dorsum with coarse, slightly sinuous longitudinal rugae; pronotum with coarse irregular rugae and reticulations; mesopleura and lower part of propodeum with longitudinal rugose. Petiolar node and postpetiole dull, more coarsely rugose than in the worker, ground sculptures developed.

**Males.** Unknown.

#### Habitat.

Found foraging on the ground of alpine meadow at the altitudes of 3437m. Nesting site unknown.

#### Etymology.

The specific epithet is the last name of a famous Chinese artist in the Ming Dynasty, Qichang Dong.

#### Differential diagnosis.

This species belongs to the *pachei* group. The worker of this group is easily distinguished from other *Myrmica* species by a combination of the following characters: mesosoma dorsum at least partly with transverse rugosity; scape gradually though distinctly curved at the base, not angled, with no trace of lobe or carina. Anterior clypeal margin rounded or slightly prominent with no medial notch; petiole with a relatively short peduncle. Radchenko and Elmes (2001) once believed that this group was only found in Himalaya. However, following the recent examination of the *Myrmica* of China (Radchenko and Elmes 2009), they found out that the *pachei* group was much more diverse than previously expected. Before this study, this group contains 15 species. Radchenko and Elmes (2009) have made a good taxonomic revision and provided a key to the group based on workers. In this study, four new species of this group are described. Because the *pachei*-group is a sizeable species group and the sole function of a key is to allow taxa to be identified, a revised key is necessary for this group of species of the Old World. Accordingly, this key is given at the end of this paper, and distinguishing morphological characters between each species in the *pachei* group is obvious. It is easy to find that *Myrmica dongi* sp. n. is very similar to *Myrmica pleiorhytida* Radchenko & Elmes, but differs from the latter by anterior surface of the petiole concave, dorsum of node with a distinct dorsal plate, slightly convex, posterior surface steep. Only the mesonotal dorsum with a fine transverse rugae, number of rugae on this area < 20, number of rugae between frontal carinae level the eyes ≤ 20.

This species is also closely related to *Myrmica dongi* sp. n., but differs from the latter by petiole with a stronger triangular ventral process; propleuron with rugose; mesonotal and propodeal dorsum with about 20 moderately coarse transverse sinuous rugae.

### Myrmica liui sp. n.

Taxon classificationAnimaliaHymenopteraFormicidae

http://zoobank.org/6AFDE2BC-646A-4E51-8AEB-FC39BEA32692

Figures 14–16

#### Type material.

**Holotype worker.** Helanshan, Inner Mongolia Autonomous Region, China, 38°52'29"N, 105°53'42"E, 2597m. 8.vii.2010, leg. Zhilin Chen, No. G100237. **Paratypes.** 5 workers, data as per holotype.

#### Measurements and descriptions.

**Holotype worker**. HL 1.13, HW 0.93, FW 0.40, FLW 0.50, SL 0.88, PW 0.65, ML 1.38, PL 0.38, PH 0.38, ESL 0.18, CI 1.21, FI 0.43, FLI 1.25, SI_1_ 0.78, SI_2_ 0.94, ESLI 0.19. **Paratype workers** (n = 6). HL 1.10−1.15, HW 0.91−0.94, FW 0.35−0.41, FLW 0.45−0.50, SL 0.85−0.90, PW 0.60−0.71, ML 1.33−1.40, PL 0.32−0.42, PH 0.33−0.41, ESL 0.13−0.17, CI 1.18−1.22, FI 0.31−0.34, FLI 1.28−1.33, SI_1_ 0.77−0.82, SI_2_ 0.90−0.96, ESLI 0.18−0.21.

**Holotype worker.** Head longer than broad, with weakly convex sides and posterior margin, and broadly rounded posterior corners; anterior clypeal margin broadly rounded, shallowly notched medially. Frontal carinae very feebly curved, merging with the rugae that extend to the margin of the head. Frons wide, frontal lobes much extended. Antennal scape long (SI_2_ 0.94), slightly shorter than head width, gradually though distinctly curved at the base, with ridge on the inner margin.

Mesosoma in profile view weakly convex; promesonotal suture in dorsal view visible, metanotal groove very weak or absent. Propodeal lobes rounded. Propodeal spines short, blunt, directly backward and downward. Petiole with anterior surface concave, meeting the dorsal one to form a blunt angle, dorsum of node somewhat convex and steeply sloping backward, without dorsal plate. Postpetiole shorter than high, with convex dorsum. Spurs on middle and hind tibiae well-developed and pectinate.

Head with coarse longitudinal rugae on the whole dorsum, number of rugae between frontal carinae level with the eyes < 20. Posterior part and lateral sides of the head coarsely reticulated. Clypeus with coarse longitudinal rugae. Frontal triangle with a few longitudinal rugae, space between rugae shiny.

Dorsum and sides of mesosoma with less sinuous longitudinal rugae, space between rugae smooth and shiny. Petiole and postpetiole with short rugae, and densely punctate.

Head with abundant, long, suberect hairs at lateral margins; dorsum of mesosoma with longer hairs, petiole with 6−8 long and some shorter hairs. Antennal scape and tibiae with subdecumbent hairs. Head and gaster colored dark brown, mesosoma reddish-brown, appendages lighter.

**Paratype workers.** As holotype.

**Queens and males.** Unknown.

#### Habitat.

This species nests in the soil in alpine meadow, at elevation 2573m.

#### Etymology.

The specific epithet is the Chinese name Gongquan Liu, who was a famous Chinese calligrapher in the Tang Dynasty.

#### Differential diagnosis.

This species belongs to the *lobicornis* species group, which is one of the three most diverse species group of the Old World, containing 22 species (Radchenko and Elmes 2010). Radchenko and Elmes (2010) divided this group into five species complex, based on worker characters. This species shares features of *kasczenkoi*-complex of this group by mesosoma with less coarse sinuous longitudinal rugosity, propodeal spines shorter (ESLI ≤ 0.35), petiole of various shape, but never with well developed flattened dorsal plate. The *kasczenkoi*-complex includes 5 species: *Myrmica angulinodis*, *Myrmica commarginata*, *Myrmica displicentia*, *Myrmica kamtschatica* and *Myrmica kasczenkoi*. *Myrmica liui* sp. n. is similar to *Myrmica angulinodis*, but the latter propodeal spines that curved inward when viwed from above. *Myrmica liui* sp. n. is very similar to *Myrmica commarginata*, but differs from the latter by dorsum of petiole somewhat convex and steeply sloping backward, without dorsal plate; on the other hand, the latter possesses unique morphological feature: mesonotum and propodeum are strongly conwtricted laterally, so that dorsal surface of them is narrow and form a sharp fidges, merging with the outer bases of propodeal spines. *Myrmica liui* sp. n. also semblables to *Myrmica displicentia*, but differs from the latter by dorsum of petiole without dorsal plate. This species is also similar to *Myrmica kasczenkoi* Ruzsky, but differs from the latter by antennal scape with ridge at the base of the inner margin; propodeal spines thin, short, only 1/2 times longer than the distance between them, somewhat narrow at the base, backward and curved downward; petiole without dorsal plate. This species resembles to *Myrmica kamtschatica*, but well differs from the latter by frontal carinae merges with the rugae that extend to the margin of the head, petiole without dorsal plate.

This species also is similar to *Myrmica sulcinodis* Nylander of the *sulcinodis*-complex, but differs from the latter by sides of petiolar node with punctures and short rugae less coarse than those on the mesosoma. Metanotal groove very weak or absent. Anterior surface of petiole concave, meeting the dorsal one through a rounded angle, dorsum of node somewhat convex and steeply sloping backward.

**Figures 14–16.**
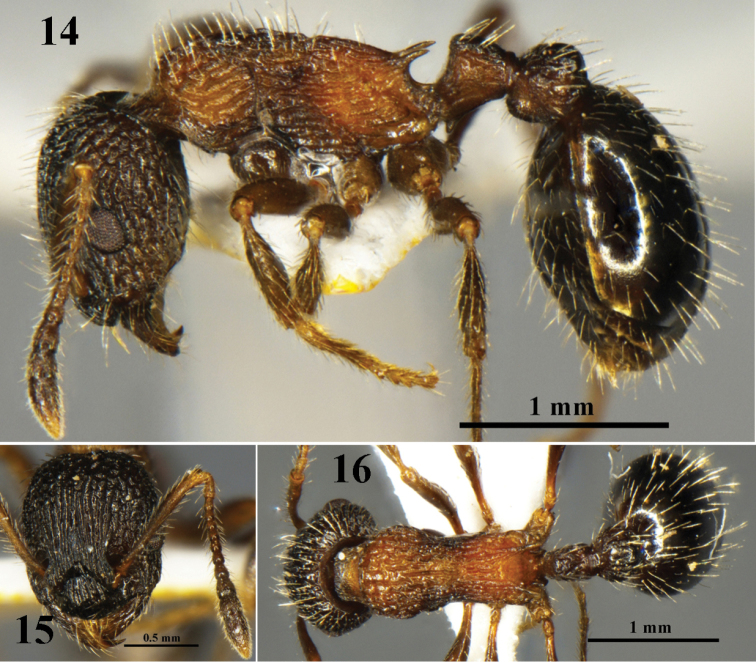
*Myrmica liui* sp. n. worker (No. G100237). **14** body in profile view **15** head in full-face view **16** body in dorsal view.

### Myrmica huaii sp. n.

Taxon classificationAnimaliaHymenopteraFormicidae

http://zoobank.org/CA7198CA-28D5-451F-87B9-541A7024CF33

Figures 17–19

#### Type material.

**Holotype worker.** Huangbaiyuan, Shaanxi Prov., China, 33°59'48"N, 107°17'42"E, 1927m. 8. vi. 2012, No. G120347, leg. Chaotai Wei, **Paratypes.** 1 worker, 2. vi. 2012, No. G1200339; 1 worker, 27. vi. 2012, No. G090467. Locality and collector the same as holotype.

#### Measurements and descriptions.

**Holotype worker**. HL 1.50, HW 1.25, FW 0.55, FLW 0.58, SL 1.25, PW 0.85, ML 1.90, PL 0.55, PH 0.43, ESL 0.45, CI 1.20, FI 0.44, FLI 1.05, SI_1_ 0.83, SI_2_ 1.00, ESLI 0.36. **Paratype workers** (n = 3). HL 1.48−1.55, HW 1.21−1.29, FI 0.50−0.55, FLW 0.50−0.57, SL 1.20−1.31, PW 0.80−0.83, ML 1.86−1.91, PL 0.50−1.57, PH 0.41−0.44, ESL 0.41−0.45, CI 1.20−1.23, FI 0.41−0.44, FLI 1.00−1.09, SI_1_ 0.80−0.83, SI_2_ 0.97−1.02, ESLI 0.35−0.37.

**Holotype worker.** Head longer than broad, with weakly convex sides and posterior margin, and narrowly rounded posterior corners; anterior clypeal margin relatively narrowly rounded, but not prominent and not notched medially. Frontal carinae very feebly curved, merging with the rugae that surround antennal sockets. Frons wide, frontal lobes not extended. Antennal scape relatively long (SI_2_ = 1.00), equal to head width, gradually curved at the base, without any trace of lobe or carina.

Mesosoma robust, promesonotum in profile view distinctly convex, promesonotal suture in dorsal view indistinct. Metanotal groove distinct, deep and abrupt. Propodeal lobes rounded. Propodeal spines straight, thin, acute, directly backward at an angle of approximately 30º. Petiole with distinct peduncle, anterior surface slightly concave, and dorsum of node broadly rounded, with a distinct dorsal plate. Postpetiole subglobular, with anterior and dorsal surfaces forming a regular arch. Spurs of middle and hind tibiae well-developed and pectinate. Frons with dense, fine, slightly sinuous longitudinal rugae, number of rugae between frontal carinae level with the eyes > 25, posterior part and lateral sides of the head with fine reticulation, space between rugae dull, densely and coarsely punctate. Clypeus with longitudinal rugae, spaces between them shiny, frontal triangle smooth and shiny.

Pronotal dorsum reticulate, lateral sides reticulate-punctate. Mesonotal and propodeal dorsum each with more than ten moderately coarse sinuous transverse rugae. Lower parts of mesopleura and sides of propedeum with longitudinal rugae. Space between rugae on mesosoma with fine punctures, though appearing quite shiny. Petiole and postpetiole dull, densely punctate and reticulated. Anterior third of first gastral tergite with fine superficial hexagonal sculpture, the rest of the tergite smooth and shiny.

Head with short subdecumbent hairs at lateral margins above the eyes, posterior part of the head without additional long hairs, genae with a few long hairs; dorsum of mesosoma with long hairs; petiole with 4−6 long and a few short hairs. Antennal scape and tibiae with decumbent hairs. Gaster with short suberect hairs. Head and gaster blackish-brown, mesosoma yellowish-brown, appendages somewhat lighter.

**Paratype workers.** As holotype, but gaster with less short suberect hairs; petiole and postpetiole middle densely punctuate and the longitudinal rugae of frons more rough than holotype.

**Queens and males.** Unknown.

#### Habitat.

Found foraging on the ground in coniferous forest at an altitude of 1927 m. Nesting site unknown.

#### Etymology.

The specific epithet is the Chinese name Su Huai, who was a famous Chinese calligrapher in the Tang Dynasty.

#### Differential diagnosis.

This species belongs to the *pachei* group. It is easy to find that this species is very similar to *Myrmica schulzi* and *Myrmica phalacra*, but differs from the latters two by basal third of first gastral tergite with fine superficial hexagonal sculpture; posterior margin without any erect to suberect long hairs; dorsum of petiolar node with a distinct broad dorsal plate. Main discriminative morphological characters with other species of the *pachei*-group is showed in the key of *pachei*-group species.

**Figures 17–19.**
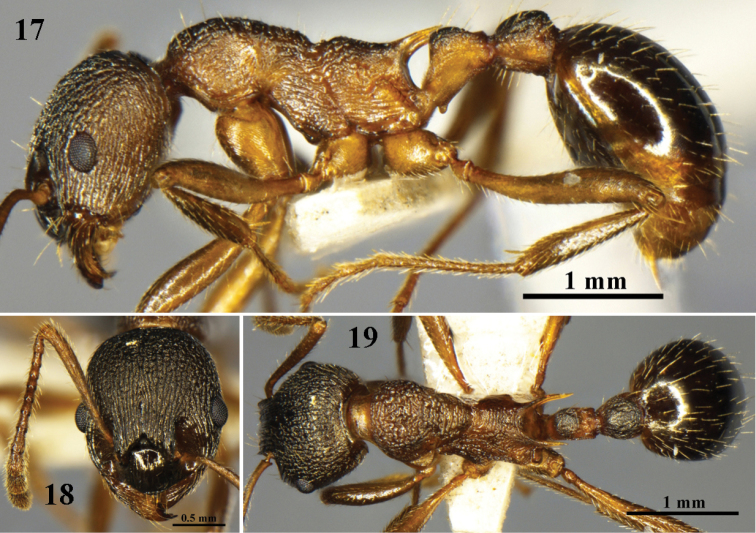
*Myrmica huaii* sp. n. worker (No. G120347). **17** body in profile view **18** head in full-face view **19** body in dorsal view.

### Myrmica mifui sp. n.

Taxon classificationAnimaliaHymenopteraFormicidae

http://zoobank.org/B65D6044-B6A6-4049-95E1-9B7B65A5FFC5

Figures 20–22

#### Type materials.

**Holotype worker.** Taibai Mt., Shaanxi Prov., China, 33°59'57"N, 107°47'17"E, 3020m. 20.viii.1997, leg. Cong Wei, No. G970018; **Paratypes.** 3 workers, as holotype.

#### Measurements and descriptions.

**Holotype worker**. HL 1.55, HW 1.38, FW 0.60, FLW 0.63, SL 1.28, PW 0.90, ML 1.95, PL 0.50, PH 0.48, ESL 0.48, CI 1.12, FI 0.43, FLI 1.05, SI_1_ 0.82, SI_2_ 0.92, ESLI 0.35. **Paratype workers** (n=2). HL 1.54−1.58, HW 1.37−1.40, FW 0.60−0.62, FLW 0.61−0.63, SL 1.25−1.26, PW 0.90−0.92, ML 1.91−1.94, PL 0.50−1.52, PH 0.50−0.51, ESL 0.46−0.49, CI 1.13−1.14, FI 0.42−0.44, FLI 1.04−1.05, SI_1_ 0.80−0.81, SI_2_ 0.90−0.93, ESLI 0.33−0.35.

**Holotype worker.** Head longer than broad, with very weakly convex sides and almost straight posterior margin, and rounded posterior corners; anterior clypeal margin rounded, slightly prominent, not notched medially. Frontal carinae very feebly curved, merging with the rugae that extend to the posterior third dorsum of head. Frons wide, frontal lobes not extended. Antennal scape relatively long, gradually though distinctly curved at the base, without any trace of lobe or carina.

Mesosoma relatively robust, promesonotum in profile view convex, promesonotal suture in dorsal view well-developed. Metanotal groove distinct, very deep. Propodeal lobes triangular apically. Propodeal spines moderately long, straight, sharp, directly backward at an angle of about 45º. Petiole high, with very short peduncle, its anterior surface slightly concave, dorsum of node with a distinct dorsal plate, slightly convex, posterior surface steep, so that petiolar node appears sharply cylindroid (seen in profile). Postpetiole subglobular, its anterior and dorsal surfaces forming a regular arch. Spurs of middle and hind tibiae well-developed and pectinate.

Head with very dense, fine, almost straight, slightly posteriorly diverging longitudinal rugae on the whole dorsum, number of rugae between frontal carinae level with the eyes < 25. Posterior part and lateral sides of the head with reticulation, surface between reticulation densely superficially punctate, appearing more or less shiny and not dull. Clypeus with longitudinal rugae, surface between them shiny. Frontal triangle smooth and shiny.

Pronotal dorsum reticulated, lateral sides reticulate-punctate; mesonotal dorsum with 8−10 coarse sinuous transverse rugae; dorsum of propodeum with several finer transverse rugae; lower parts of mesopleura and sides of propodeum with fine longitudinal rugae. Space between rugae on mesosoma smooth and shiny. Petiole high, and with a strongly triangular ventral process. Petiole and postpetiole with short irregular rugae, densely though not coarsely punctate, appearing dull.

Margins of head with long suberect hairs; dorsum of mesosoma with longer hairs, petiole with 6−8 long hairs. Antennal scape and tibiae with subdecumbent hairs. Body colored blackish-brown, appendages somewhat lighter.

**Paratype workers.** as holotype.

**Queens and males.** Unknown.

#### Habitat.

Found in mountain meadows at an altitude of 3020 m. Nesting site unknown.

#### Etymology.

The specific epithet is the name of a famous calligrapher in the Northern Song Dynasty.

#### Differential diagnosis.

This species belongs to the *pachei* group. It is easy to find that this species is very similar to *Myrmica pleiorhytida*, but differs from the latter by number of rugae between frontal carinae level with the eyes ≤ 25; mesonotal and propodeal dorsum fine transverse rugae < 20. This species also very resemles to *Myrmica dongi* sp. n., but differs from the latter by petiole with a finer triangular ventral process; propleuron only with densely punctuated; mesonotal and propodeal dorsum with 8−10 coarse sinuous transverse rugae. Main discriminative morphological characters with other species of the *pachei*-group is showed in the key of *pachei*-group species.

**Figures 20–22.**
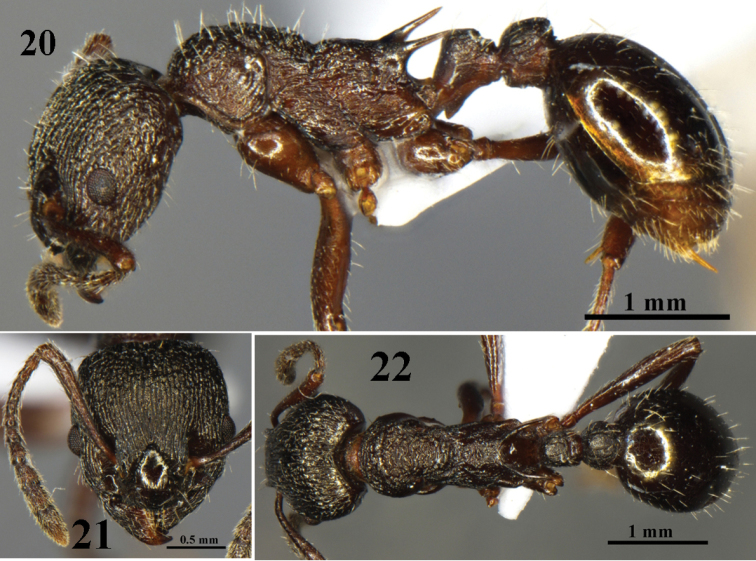
*Myrmica mifui* sp. n. worker (No. G970018). **20** body in profile view **21** head in full-face view **22** body in dorsal view.

### Myrmica oui sp. n.

Taxon classificationAnimaliaHymenopteraFormicidae

http://zoobank.org/D6CD7B14-D6CF-4F48-A611-749B390D3E7E

Figures 23–25

#### Type material.

**Holotype worker.** Kuankuoshui, Guizhou Prov., China, 28°14'24"N, 107°12'00"E, 1202m. 16.viii.2010, leg. Duoduo Ye, No. G100231. **Paratypes.** 4 workers, data as holotype.

#### Measurements and descriptions.

**Holotype worker.** HL 1.50, HW 1.32, FW 0.53, FLW 0.55, SL 1.60, PW 1.00, ML 2.25, PL 0.63, PH 0.40, ESL 0.68, CI 1.14, FI 0.40, FLI 1.04, SI_1_ 1.07, SI_2_ 1.21, ESLI 0.51. **Paratype workers** (n = 2). HL 1.44−1.50, HW 1.28−1.33, FI 0.40−0.43, FLI 1.04−1.06, SL 1.56−1.59, PW 1.00−1.08, ML 2.10−2.17, PL 0.63−0.67, PH 0.40−0.45, ESL 0.63−0.64, CI 1.10−1.18, FW 0.50−0.59, FLW 0.50−0.56, SI_1_ 1.02−1.08, SI_2_ 0.77−0.80, ESLI 0.50−0.55.

**Holotype worker.** Head longer than broad, with very feebly convex sides, nearly straight posterior margin and broadly rounded posterior corners. Anterior clypeal margin very feebly convex, notched medially. Frontal carinae curved, merging with the rugae that extend to the posterior third dorsum of head. Frons wide, frontal lobes not extended, but raised vertically (i.e. perpendicular to the surface of the head). Antennal scape relatively long (SI_2_ 1.21), longer than head width, gradually though distinctly curved at the base, without any trace of lobe or carina.

Mesosoma relatively short (compared to related species), promesonotal dorsum in profile view finely convex, promesonotal suture in dorsal view indistinct; mesonotum abruptly curved down to propodeum to form distinct, deep and wide metanotal groove. Propodeal lobes projecting to form short and blunt triangle. Propodeal spines relatively long, widened at the base, directly backward and downward. Petiole relatively long and narrow, with strongly concave of anterior surface, dorsum of node feebly convex, with distinct dorsal plate; postpetiole as shown in figures, slightly shorter than high.

Head with fine, almost straight, posteriorly diverging longitudinal rugae on the whole dorsum, eight rugae between frontal carinae level with the eyes. Posterior part and sides of the head without reticulations, spaces between rugae densely punctate, dull. Clypeus with longitudinal rugae, surface between rugae shiny. Frontal triangle smooth and shiny.

Dorsum of mesosoma with coarse reticulation, lateral sides with coarse sinuous longitudinal rugae. Lower part of mesopleuron and sides of propodeum with coarse longitudinal rugae. In dorsal view, dorsum of propodeal behind the metanotal groove with a distinct U-shaped coarse rugae (seen in Fig. 25). Petiole and postpetiole at most with very fine sculptures or short irregular rugae and dense, though not coarse, punctures and dull.

Head posterior margin with long suberect hairs; mesosoma dorsum with longer hairs, petiole with 6−8 long hairs. Antennal scape with suberect hairs. Tibiae with subdecumbent hairs. Head, gaster and petiole and postpetiole brownish-red, dorsum of head with some dark patches. Mesosoma black to blackish-brown.

**Paratype workers.** As holotype, but in one individual, petiole only with 4 long hairs.

**Queens and males.** Unknown.

#### Habitat.

This species nests under litter layer and soil layer in the broadleaf forests, at elevation 1202m.

#### Etymology.

The specific epithet is the last name of a famous Chinese artist in the Tang Dynasty, Yanxun Ou.

#### Differential diagnosis.

This species belongs to the *draco*-complex of the *ritae* species group that includes *Myrmica draco*, *Myrmica plodii*, *Myrmica schoedli*, *Myrmica yamanei*. The workers of this species complex seems to be intermediate between the *ritae*-complex and boltoni-complex, but differs from the latter two by head dorsum and mesosoma rugose, petiole and postpetiole finely striated and punctuated, space of head dorsum between rugae punctuated. In terms of geography, *Myrmica oui* sp. n. and *Myrmica draco* may be occupying similar niches, but former differs from the latter by mesonotum abruptly curving down to the propodeum to form a distinct, deep and wide metanotal groove; in dorsal view, the dorsum of propodeum behind the metanotal groove bears a distinct U−shaped coarse ruga; first gastral tergite with clear superficial hexagonal microsculpture; body large (HW=1.38), dorsum of head with some dark patches. Given these obvious morphological differences, we are certain that *Myrmica oui* sp. n. is not a variety of *Myrmica draco* but an independent science species.

**Figures 23–25.**
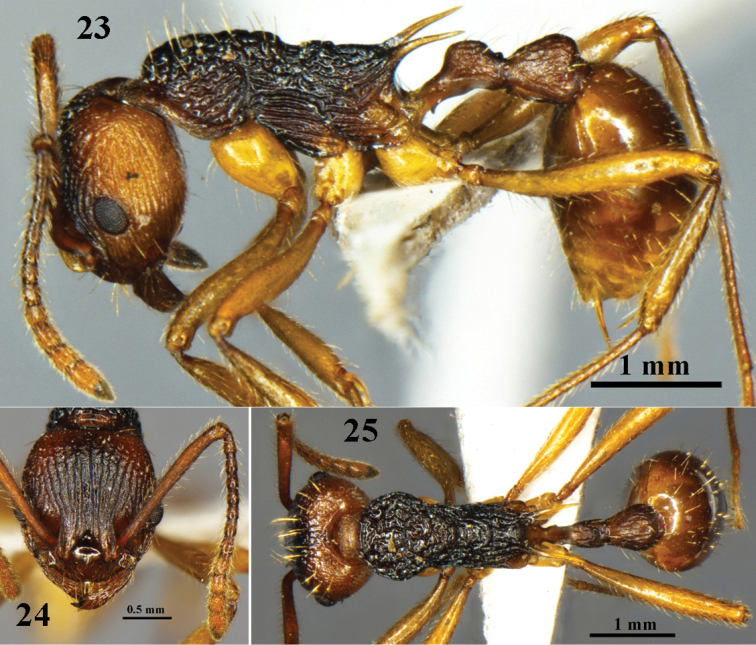
*Myrmica oui* sp. n. worker (No. G100231). **23** body in profile view **24** head in full-face view **25** body in dorsal view.

### Myrmica wangi sp. n.

Taxon classificationAnimaliaHymenopteraFormicidae

http://zoobank.org/256A0E94-3A7C-4528-B4AE-2DBA33986625

Figures 26–28

#### Type material.

**Holotype worker.** Huangbaiyuan, Shaanxi Prov., China, 34°10'36"N, 107°11'03"E, 1567m. 1.vi.2012, leg. Chaotai Wei, No. G120127. **Paratypes.** 5 workers, data as holotype.

#### Measurements and descriptions.

**Holotype worker**. HL 1.62, HW 1.42, FW 0.55, FLW 0.58, SL 1.67, PW 1.05, ML 2.25, PL 0.63, PH 0.43, ESL 0.75, CI 1.14, FI 0.39, FLI 1.05, SI_1_ 1.03, SI_2_ 1.18, ESLI 0.53. **Paratype workers** (n = 5). HL 1.60−1.67, HW 1.33−1.41, FW 0.54−0.56, FLW 0.57−0.60, SL 1.56−1.64, PW 1.00−1.11, ML 2.19−2.23, PL 0.59−0.64, PH 0.40−0.44, ESL 0.70−0.77, CI 1.13−1.17, FI 0.38−0.41, FLI 1.02−1.08, SI_1_ 1.00−1.04, SI_2_ 1.17−1.20, ESLI 0.51−0.54.

**Holotype worker.** Head longer than broad, with very feebly convex sides, nearly straight posterior margin and broadly rounded posterior corners. Anterior clypeal margin very feebly convex, notched medially. Frontal carinae very feebly curved, merging with the rugae that extend to the posterior third dorsum of head. Frons wide, frontal lobes not extended, but raised vertically (i.e. perpendicular to the surface of the head). Antennal scape relatively long (SI_2_ = 1.18), longer than head width, gradually though distinctly curved at the base, without any trace of lobe or carina.

**Figures 26–28.**
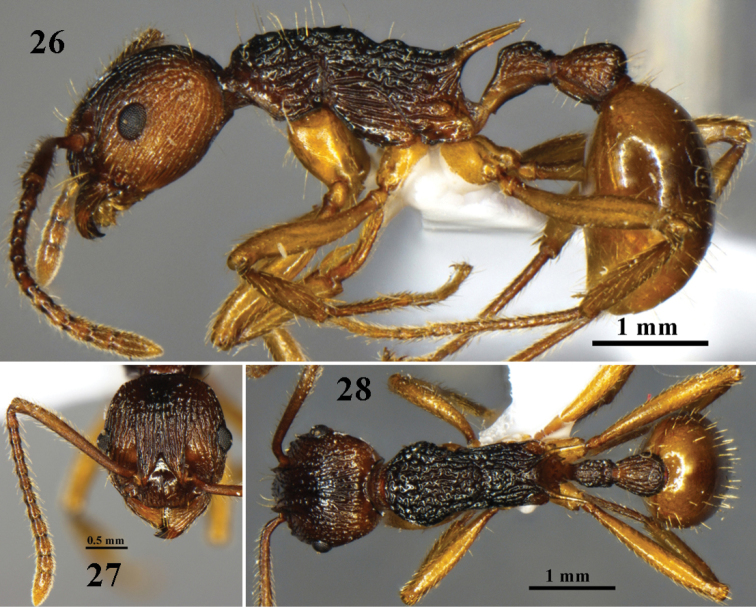
*Myrmica wangi* sp. n. worker (No. G120127). **26** body in profile view **27** head in full-face view **28** body in dorsal view.

Promesonotal dorsum in profile view convex, promesonotal suture in dorsal view indistinct; mesonotum abruptly curved down to propodeum to form distinct, deep and wide metanotal groove. Propodeal lobes projecting to form short blunt triangle. Propodeal spines relatively long, widened at the base, directly backward and slightly downward. Petiole relatively short and wide, with anterior surface strongly concave, dorsum of node feebly convex; postpetiole somewhat shorter than high (Fig. 26).

Head with fine, almost straight, posteriorly diverging longitudinal rugae on the whole dorsum extending back to posterior margin, eight rugae between frontal carinae level with the eyes. Posterior part of the head with reticulations, space between rugae finely superficially micro−punctate. Clypeus with longitudinal rugae, space between them shiny. Frontal triangle smooth and shiny.

Dorsum of mesosoma with coarse reticulation, lateral sides with coarse sinuous longitudinal rugae. Petiole with coarse, short, sinuous longitudinal rugae, postpetiole with less coarse longitudinal, slightly sinuous rugae. Space on body between rugae smooth and shiny.

Posterior margin of head with up to two long suberect hairs; mesosoma dorsum with longer hairs, petiole with 1−6 long hairs. Antennal scape with suberect hairs. Tibiae with subdecumbent hairs. Head, gaster and petiole and postpetiole brownish-red, mesosoma black to blackish-brown.

**Paratype workers.** As holotype.

**Queens and males.** Unknown.

#### Habitat.

This species nests inside decayed wood in the broadleaf and coniferous forests, at elevation 1667m.

#### Etymology.

The specific epithet is the last name of a famous Chinese artist in the Eastern Jin Dynasty, Xizhi Wang.

#### Differential diagnosis.

*Myrmica wangi* sp. n. belongs to the *draco*-complex of the *ritae* species group. This species group includes 5 species: *Myrmica draco*, *Myrmica oui* sp. n., *Myrmica plodii*, *Myrmica schoedli*, *Myrmica yamanei*. So far, only two species (*Myrmica draco* and *Myrmica wangi* sp. n.) of the *ritae* species group were found from Shaanxi Province, which is the highest latitude distribution areas of this species group in the Old world. We investigated the two paratypes workers of *Myrmica draco* Radchenko, Zhou & Elmes found that two species are very similar to each other, but *Myrmica wangi* sp. n. differs from the *Myrmica draco* by the nearly straight posterior margin and broadly rounded posterior corners, frontal carinae extend back to posterior margin, posterior part of the head without reticulation; only posterior margins with 0−2 long suberect hairs; propodeal lobes projecting to form short and blunt triangle; petiole with coarse, short, sinuous longitudinal rugae, petiole and postpetiole with fewer punctures, appears shiny. On the other hand, This species is also similar to *Myrmica oui* sp. n., but differs from the latter by the surface between rugae on the head with fewer punctures and appearing shiny. In dorsal view, dorsum of propodeum behind the metanotal groove with irregular coarse rugae. Petiole and postpetiole with coarse, short, sinuous longitudinal rugae, with fewer punctures, appearing shiny. We considered that these morphological differences is very obvious, which could be easily aparted from the other species of the genus *Myrmica*.

### Myrmica yani sp. n.

Taxon classificationAnimaliaHymenopteraFormicidae

http://zoobank.org/1B9C924D-1F59-43E5-8D4A-609E2A46481E

Figures 29–34

#### Type material.

**Holotype worker.** Fanjingshan Nature Reserve, Guizhou Prov., China, 27°54'26" N, 108°38'44"E, 1667m. 30.v.2002, leg. Shanyi Zhou, No. G020318. **Paratypes.** 3 workers and 1 queen, data as holotype.

#### Measurements and descriptions.

**Figures 29–34.**
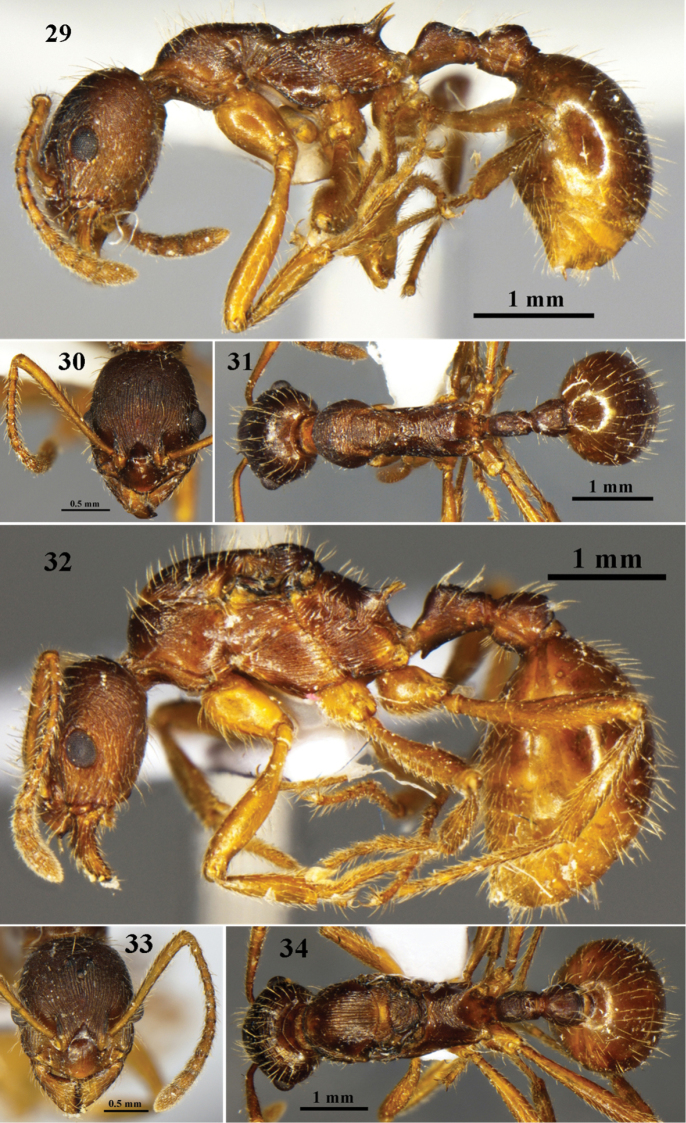
*Myrmica yani* sp. n. **29−31** Worker (G020318) **32−34** Queen (G020318). **29, 32** body in profile view **30, 33** head in full-face view **31, 34** body in dorsal view.

**Holotype worker** (Figs 29–31). HL 1.53, HW 1.18, FW 0.53, FLW 0.55, SL 1.50, PW 0.85, ML 2.08, PL 0.55, PH 0.25, ESL 0.33, CI 1.30, FI 0.45, FLI 1.04, SI_1_ 0.98, SI_2_ 1.27, ESLI 0.28. **Paratype workers** (n = 15). HL 1.48−1.52, HW 1.17−1.23, FW 0.52−0.54, FLW 0.53−0.57, SL 1.38−1.43, PW 0.81−1.86, ML 2.11−2.14, PL 0.53−0.59, PH 0.21−0.27, ESL 0.30−0.37, CI 1.28−1.31, FI 0.43−0.46, FLI 1.02−1.06, SI_1_ 0.98−1.03, SI_2_ 1.25−1.27, ESLI 0.27−0.29. **Paratype Queen** (Figs 32–34). HL 1.63, HW 1.33, FW 0.60, FLW 0.63, SL 1.50, PW 1.13, ML 2.50, PL 0.68, PH 0.53, ESL 0.25, CI 1.26, FI 0.45, FLI 1.05, SI_1_ 0.92, SI_2_ 1.27, ESLI 0.19.

**Holotype worker.** Head longer than broad, with weakly convex sides and posterior margin, and narrowly rounded posterior corners; anterior clypeal margin narrowly rounded, not notched medially. Frontal carinae very feebly curved, merging with the rugae that extend to the posterior half dorsum of head. Frons wide, frontal lobes not extended. Antennal scape relatively long (SI_2_ 1.27), gradually curved at the base, without any trace of lobe or carina.

Promesonotum in profile view slightly convex, promesonotal suture in dorsal view indistinct. Metanotal groove distinct, deep and abrupt. Propodeal lobes rounded. Propodeal spines quite short, straight, thin, acute, directly backward at an angle of about 30º. Petiole with distinct, but short peduncle, anterior surface slightly convex, meeting the dorsal one to form a blunt, narrowly rounded angle, dorsal surface short, gradually sloping posteriorly, without dorsal plate. Postpetiole subglobular, anterior and dorsal surfaces forming a feeble arch. Spurs of middle and hind tibiae well-developed and pectinate. Frons with dense, fine, slightly sinuous longitudinal rugae, number of rugae between frontal carinae level with the eyes is >20; posterior third dorsum of head densely punctate; posterior part of the head densely micropunctate and dull. Clypeus almost smooth, at most with some fine longitudinal rugae, space between rugae shiny. Frontal triangle smooth and shiny.

Mesosoma with fine transverse rugae in the whole of dorsum, space between rugae with micropunctures and dull. Posterior of petiole with fine short rugae, the rest of petiole and postpetiole densely punctate, appearing dull.

Head with abundant long hairs at margins, genae with a few long hairs; dorsum of mesosoma with long hairs; petiole with 4−6 long hairs and a few short hairs. Antennal scape and tibiae with subdecumbent hairs. Body colored yellowish brown, appendages somewhat lighter.

**Paratype workers.** With similar morphological characters as holotype, but in one individual, dorsum of mesonotum and front part of pronotum of transverse rugae is abscure.

**Paratype queen.** Queen generally similar to workers by the shape and sculptures of head (except posterior dorsum of head with fine transverse rugae), frontal lobes, propodeal spines and petiole and postpetiole. Anterior half of scutum with sinuous longitudinal rugae and reticulation; scutum with coarse longitudinal rugae, scutellum concentrically rugulose, propodeal dorsum with transverse rugae; lateral of mesosoma with slightly less coarse longitudinal rugae. Petiolar node and postpetiole with some irregular rugae, space between rugae densely punctate, appearing dull.

**Males.** Unknown.

#### Habitat.

This species nests inside decayed wood in the broadleaf and coniferous forests, at elevation 1667m.

#### Etymology.

The specific epithet is the last name of a famous Chinese artist in the Tang Dynasty, Zhenqing Yan.

#### Differential diagnosis.

*Myrmica yani* sp. n. is a remarkable new species, belonging to the *pachei* group. So far, only three species (*Myrmica pachei*, *Myrmica inezae* and *Myrmica villosa*) are recorded from the Himalayas which possess the key character of the whole mesosoma of dorsum bearing transverse rugae. *Myrmica yani* sp. n. differs from the *Myrmica pachei* and *Myrmica inezae* by having a distinctly elongated head, with narrowly rounded posterior corners; posterior third of head dorsally without longitudinal rugae and reticulation, but densely punctate; petiole with distinct, short peduncle, its anterior surface slightly convex, meeting the dorsal one to form a blunt, narrowly rounded angle; dorsal surface short, gradually sloping posteriorly; body colored yellowish brown. It differs from *Myrmica villosa* by the distinctly elongated head, with narrowly rounded posterior corners; posterior third of head dorsally without longitudinal rugae, but densely punctate; propleuron with densely micropunctures and dull; dorsum of propodeum with fine transverse rugae; anterior surface of petiole slightly convex, meeting the dorsal one to form a blunt, narrowly rounded angle.

### Key to *Myrmica* species found in China based on the worker caste

*The key is modified from Radchenko and Elmes (2010). Any doubtful species are excluded here; *Myrmica mixta* is also excluded from the key because the worker caste is not well-known.

**Table:** 

1	Lateral portion of clypeus raised into a sharp ridge in front of the antennal insertions, so that the antennal sockets are distinctly separated from the clypeal surface (similar to that of *Tetramorium*) (Radchenko and Elmes 2010: fig. 80, A)	**2**
−	Lateral portion of clypeus not raised into a sharp ridge in front of the antennal insertions, so that the antennal sockets lay on the same level with the clypeal surface (Radchenko and Elmes 2010: fig. 134, A)	**3**
2	Antennal scape at the bend having the same width as at its mid−length; the base of the scape having a longitudinal groove and lateral ridges (Radchenko and Elmes 2010: figs 80, A–E)	***Myrmica excelsa* Kupyanskaya**
−	Antennal scape at the bend distinctly narrower than at its mid−length; the base of the scape without longitudinal groove and lateral ridges (Radchenko and Elmes 2010: figs 290, A–E)	***Myrmica transsibirica* Radchenko**
3	Frontal carinae curved outward to merge with the rugae that surround the antennal socket (Radchenko and Elmes 2010: figs 161, A; 213, A). Antennal scape very smoothly curved at the base, not angled and without any trace of lobe or carina (Radchenko and Elmes 2010: figs 161, B; 213, B)	**4**
−	Frontal carinae merging with the rugae that extend to the posterior margin, not curved outward to merge with the rugae that surround the antennal socket (Radchenko and Elmes 2010: fig. 134, A). Antennal scape strongly curved or gradually curved at the base, with or without a lobe, ridge or carina (Radchenko and Elmes 2010: fig. 5, B; 22, B; 66, B; 123, B; 128, B; 134, B–E; 195, B; 239, B–C; 273, B–C; 325, B–C)	**18**
4	First gastral tergite finely but distinctly longitudinally striated. Spurs on tibiae of the middle and the hind legs reduced, simple (Radchenko and Elmes 2010: figs 161, A–E)	***Myrmica luteola* Kupyanskaya**
–	First gastral tergite smooth and shiny. Spurs on tibiae of the middle and the hind legs well-developed and pectinate	**5**
5	Mesonotal dorsum partly with transverse rugae (Radchenko and Elmes 2010: figs 250, C–D; 324, C–D)	**6**
−	Mesonotal dorsum with various sculpture, but never with transverse rugae (Radchenko and Elmes 2010: figs 58, C–D; 211, C–D)	**11**
6	Lateral margins of head either without pilosity or with short (≤0.03mm) decumbent hairs; if any long erect to suberect hairs present, then restricted to the posterior margin and genae (Radchenko and Elmes 2010: figs 201, A; 250, A; 277, A)	**7**
−	Lateral and posterior margins of head with numerous long (≥0.07mm) erect to suberect hairs (Radchenko and Elmes 2010: figs 198, A; 314, A; 324, A)	**9**
7	Propodeal spines long, ESLI > 0.45, sides of pronotum with longitudinal rugae (Radchenko and Elmes 2010: figs 277, A–E)	***Myrmica taibaiensis* Wei, Zhou & Liu**
−	Propodeal spines shorter, ESLI < 0.41, sides of pronotum finely and densely punctate	**8**
8	Basal third of the first gastral tergite with superficial hexagonal fine sculpture. Posterior margin without any erect to suberect long hairs; dorsum of petiolar node with a distinct broad dorsal plate (figs 17–19).	***Myrmica huaii* sp. n.**
−	First gastral tergite smooth and shiny; posterior margin with abundant short erect to suberect long hairs; dorsum of node quite narrowly rounded (Radchenko and Elmes 2010: figs 290, A–E)	***Myrmica schulzi* Radchenko & Elmes**
9	Head distinctly elongate, CI >1.20, suboval, with barely marked posterior corners (Radchenko and Elmes 2010: figs 102, A–E)	***Myrmica heterorhytida* Radchenko & Elmes**
−	Head slightly longer than broad, CI <1.15, nearly square, with distinctly marked posterior corners (Radchenko and Elmes 2010: fig. 198, A).	**10**
10	Anterior surface of the petiole almost straight, dorsum of node broadly rounded. Mesonotal and propodeal dorsum with finer transverse rugae, number of rugae on this area >25, number of rugae between frontal carinae level the eyes >30. Gaster with very fine superficial microsculpture (Radchenko and Elmes 2010: figs 198, A–E)	***Myrmica pleiorhytida* Radchenko & Elmes**
−	Anterior surface of the petiole concave, dorsum of node with a distinct dorsal plate, slightly convex, posterior surface steep. Only mesonotal dorsum with fine transverse rugae, number of rugae on this area < 20, number of rugae between frontal carinae level the eyes ≤ 20. Gaster smooth and shiny (Figs 8–13)	***Myrmica dongi* sp. n.**
11	One of the largest known *Myrmica* species, HW >1.60, AL >2.50. Very hairy, number of standing hairs on petiolar node > 20 (Radchenko and Elmes 2010: figs 167, A–E)	***Myrmica mirabilis* Elmes & Radchenko**
−	Smaller species, HW < 1.25, AL < 2.30 mm. Less hairy, number of standing hairs on petiolar node < 15 (Radchenko and Elmes 2010: figs 58, C–D).	**12**
12	Masticatory margin of the mandible with 11−13 teeth (Radchenko and Elmes 2010: figs 58, A–E)	***Myrmica curiosa* Radchenko & Elmes**
−	Masticatory margin of the mandible with ≤ 9 teeth	**13**
13	Petiolar node with rounded dorsum, completely without a dorsal plate, node of the petiole and the postpetiole smooth, at most very finely striated. Pronotal dorsum with sinuous longitudinal rugae, never with coarse reticulation (Radchenko and Elmes 2010: figs 213, C–D)	**14**
−	Petiolar node with a dorsal plate developed to various extents (except for *Myrmica arisana*), node of the petiole and the postpetiole with coarse sculpture and rugae; pronotal dorsum with coarse reticulation (Radchenko and Elmes 2010: figs 25, C–D; 137, C–D; 218, C–D)	**16**
14	Petiole and postpetiole almost smooth, with few punctures (Radchenko and Elmes 2010: figs 213, A–E)	***Myrmica rubra* (Linnaeus)**
−	Petiole and postpetiole with rugae or dense punctures	**15**
15	Antennal scape longer (SI_2_ > 0.93), with more abundant suberect hairs (Radchenko and Elmes 2010: figs 31, A–E)	***Myrmica bactriana* Ruzsky**
−	Antennal scape shorter (SI_2_ < 0.91), with less abundant subdecumbent hairs (Radchenko and Elmes 2010: figs 229, A–E)	***Myrmica ruzskyana* Radchenko & Elmes**
16	Petiolar node with rounded dorsum, completely without a dorsal plate, node of petiole smooth, at most very finely striated (Radchenko and Elmes 2010: figs 25, A–E)	***Myrmica arisana* Wheeler**
−	Petiolar node with a dorsal plate, node of petiole with quite coarse striated	**17**
17	Petiolar node with a distinct, sharply flattened dorsal plate, and having quite coarse, short sinuous longitudinal rugae on the lateral parts (Radchenko and Elmes 2010: figs 218, A–E)	***Myrmica ruginodis* Nylander**
−	Dorsum of the petiolar node slightly convex, dorsal plate not sharply flattened, lateral surfaces with finer short longitudinal rugae (Radchenko and Elmes 2010: figs 137, A–E)	***Myrmica kotokui* Forel**
18	Dorsum of the mesosoma entirely or partially with transverse rugae (Radchenko and Elmes 2010: figs 186, C–D; 324, C–D)	**19**
−	Dorsum of the mesosoma with various sculptures, but never with transverse rugae (Radchenko and Elmes 2010: figs 270, C–D; 303, C–D)	**27**
19	Dorsum of the mesosoma entirely with fine transverse rugae (figs 29–34)	***Myrmica yani* sp. n.**
−	Dorsum of the mesosoma partially with transverse rugae	**20**
20	Lateral and posterior margins of the head with numerous long erect to suberect hairs	**21**
−	Lateral margins of the head either without pilosity or with short decumbent hairs; if any long erect to suberect hairs present, then restricted to the posterior margin and genae	**24**
21	Mesonotal and propodeal dorsum with finer transverse rugae, number of rugae on this area ≥ 25 (Radchenko and Elmes 2010: figs 324, A–E)	***Myrmica yunnanensis* Radchenko & Elmes**
−	Mesonotal and propodeal dorsum with relatively coarser transverse rugae, number of rugae on this area ≤ 20	**22**
22	Head slightly longer than broad, CI < 1.15, nearly square, with distinctly marked posterior corners. Petiole with a stronger triangular ventral process (figs 20–22)	***Myrmica mifui* sp. n.**
−	Head distinctly elongate, CI > 1.20, suboval, with barely marked posterior corners. Petiole ventral process normal	**23**
23	Petiole low, PI_1_ 1.68, its node with elongate flattened dorsum. Body color reddish brown (Radchenko and Elmes 2010: figs 314, A–E)	***Myrmica weii* Radchenko & Zhou**
−	Petiole higher, PI_1_ < 1.55, its node with a short, slightly convex, gradually sloping posteriorly dorsum. Body color blackish brown (Radchenko and Elmes 2010: figs 169, A–E)	***Myrmica multiplex* Radchenko & Elmes**
24	Basal third of first gastral tergite densely punctate and longitudinally ruguloso−striated; this Basal third of first gastral tergite densely punctate and longitudinally rugulose-striate; this sculpture gradually petering out posteriorly, the rest of the surface of first tergite with well visible superficial hexagonal microsculpture (Radchenko and Elmes 2010: figs 250, A–E)	***Myrmica sculptiventris* Radchenko & Elmes**
−	Whole surface of first gastral tergite smooth	**25**
25	Head dorsum posterior to the eyes with reticulation, rest of head dorsum with longitudinal rugosity (Radchenko and Elmes 2010: figs 201, A–E)	***Myrmica polyglypta* Radchenko & Rigato**
−	Head dorsum posterior to the eyes with longitudinal rugosity; reticulation, if present, restricted to temples and/or posterior part of occiput	**26**
26	Rugosity on the head dorsum partly reduced. Frons level with the eyes with < 15 fine, slightly sinuous longitudinal rugae, only some of them running unbroken to the posterior margin. Propodeal dorsum with transverse rugosity. Scape longer than head width (Radchenko and Elmes 2010: figs 193, A–E)	***Myrmica phalacra* Radchenko & Elmes**
−	Rugosity on the head dorsum not reduced. The frons level with the eyes with > 20 longitudinal rugae that run unbroken to the posterior margin; surface between rugae very finely superficially punctate but appearing more or less shiny. Propodeal dorsum with short, slightly sinuous longitudinal rugae. Scape shorter than head width (Radchenko and Elmes 2010: figs 106, A–E)	***Myrmica hlavaci* Radchenko & Elmes**
27	Antennal scape strongly angled at the base, with horizontal lobe, or a vertical lobe (that can be inclined anteriorly), or denticles	**28**
−	Antennal scape gradually curved or angled at the base, never with a vertical, or inclined lobe, or denticles	**37**
28	Antennal scape strongly angled at the base, with horizontal lobe	**29**
−	Antennal scape strongly angled at the base, with a vertical lobe (that can be inclined anteriorly), or with denticles	**31**
29	Hairy species, petiole with more than 10 (usually with 12−20) long, thin and often curved hairs. Dorsum of the mesosoma entirely with longitudinal, slightly sinuous rugae, without reticulation; dorsum of the postpetiole with partly reduced sculptures. Anterior clypeal margin shallowly but distinctly notched medially. Spurs on the middle and the hind tibiae at least partly reduced and usually not pectinate (Radchenko and Elmes 2010: figs 303, A–E).	***Myrmica vandeli* Bondroit**
−	Less hairy species, petiole with less than 10 (usually not more than 8) long, straight, thick hairs. Dorsum of the mesosoma with strong sinuous longitudinal rugae and often with reticulation; dorsum of the postpetiole with coarse sculptures. Anterior clypeal margin not notched medially. Spurs on the middle and the hind tibiae as a rule well-developed and pectinate.	**30**
30	Frontal lobes less expanded, mean FLI 1.31. Antennal scape at the base with narrow horizontal ridge or at most with very small carina. Propodeal spines short (mean ESLI 0.31), not widened at the base, thin, often needle−like; metanotal groove weak or completely absent; petiolar node without dorsal plate, usually rounded, with posterior surface gradually declines to the postpetiole; sides of mesosoma with relatively coarse, regular, almost straight longitudinal rugae. Body color rather dark, brownish-red. Tibiae and tarsi with short subdecumbent hairs (Radchenko and Elmes 2010: figs 270, A–E)	***Myrmica stangeana* Ruzsky**
−	Frontal lobes more expanded, mean FLI > 1.40. Antennal scape at the base with more developed, but never massive, horizontal carina or lobe. Propodeal spines longer (means ESLI > 0.35), usually widened at the base (more thorn-like), never needle-like; metanotal groove well-developed, often deep; petiolar node with various shape; sides of mesosoma with less coarse sinuous longitudinal rugae. Body color lighter, usually ochreous or yellowish-red. Tibiae and tarsi with various hairs (Radchenko and Elmes 2010: figs 324, A–E)	***Myrmica scabrinodis* Nylander**
31	Propodeal spines thick and blunt, directed backward and upwards, and strongly curved inward; petiole with very short peduncle, anterior surface steep, meeting the dorsal one through an acute angle, so that the petiolar node appearing sharply angled in profile (Radchenko and Elmes 2010: figs 86, A–E)	***Myrmica forcipata* Karawajew (new Chinese record)**
−	Propodeal spines thin and sharp apically, not curved inward; petiole with various shapes (seen in profile), but if sharply angled, then the minimum distance between the frontal carina narrower (FI < 0.32 *vs* > 0.35 in *Myrmica forcipata*)	**32**
32	Lobe of the antennal scape forming shield−like dorsal plate along the basal surface of the scape	**33**
−	Lobe of the antennal scape not forming shield−like dorsal plate along the basal surface of the scape	**36**
33	Dorsum of head with sinuous longitudinal rugae, never with reticulation. Petiolar node and postpetiole densely punctuated (Radchenko and Elmes 2010: figs 258, A–E)	***Myrmica sinoschencki* Radchenko & Elmes**
−	Dorsum of head with reticulation at the posterior part. Petiolar node and postpetiole finely superficially punctate	**34**
34	Frontal carinae strongly curved, minimum distance between the frontal carinae narrower, FI < 0.26. Metanotal groove deep (Radchenko and Elmes 2010: figs 242, A–E).	***Myrmica schencki* Viereck**
−	Frontal carinae less curved, minimum distance between the frontal carinae wider, FI > 0.29. Metanotal groove shallow	**35**
35	Mesosoma with almost straight longitudinal rugae. Frontal lobes less extended, FLI 1.36−1.52, mean 1.41. Propodeal spines short, ESLI 0.18−0.30, mean 0.24. Body color usually dark reddish brown (Radchenko and Elmes 2010: figs 60, A–E)	***Myrmica deplanata* Emery**
−	Mesosoma with sinuous longitudinal rugae or reticulation. Frontal lobes more extended, FLI 1.50−1.67, mean 1.60. Propodeal spines longer, ESLI 0.27−0.34, mean 0.31. Mesosoma yellowish−brown, head reddish brown, gaster dark brown (Radchenko and Elmes 2010: figs 134, A–E)	***Myrmica koreana* Elmes, Radchenko & Kim**
36	Antennal scape with small denticles or an even ridge at the base. Petiole in profile with an almost straight, steep anterior face, node with a posteriorly-inclined dorsal plate, appearing subtriangular (Radchenko and Elmes 2010: figs 236, A–E).	***Myrmica saposhnikovi* Ruzsky**
−	Antennal scape with small but distinct lobe at the base. Petiole in profile with concave anterior face, node with a flattened or somewhat convex dorsal plate (Radchenko and Elmes 2010: figs 75, A–E)	***Myrmica eidmanni* Menozzi**
37	Propodeal spines long, ESLI > 0.45. Propodeal lobes pointed or blunt apically, but never rounded	**38**
−	Propodeal spines shorter, ESLI < 0.40. Propodeal lobes rounded apically	**48**
38	Surface between rugae on the head and the dorsal surface of the petiole and postpetiole shiny and smooth or at most very superficially micropunctate; head, mesosoma, petiole and postpetiole usually with similar coarse rugae	**39**
−	Surface between rugae on the petiole and postpetiole dull, always distinctly and often coarsely punctate; surface of head usually with similar punctures (except for *Myrmica angulata*), rugae on dorsum of the head often noticeably finer than that on the mesosoma	**44**
39	Dorsum of head entirely with almost straight, subparallel rugae, completely lacking reticulation, more than 6 rugae between frontal carinae level with the eyes	**40**
−	Dorsum of head with distinctly sinuous rugae and reticulation, if reticulation not developed, less than 6 rugae between frontal carinae level with the eyes.	**41**
40	Seen at magnification x l00, fine sculpture on surface of the head completely invisible. Pronotum with longitudinal rugae. Head yellow, strongly contrasting with the darker mesosoma (Radchenko and Elmes 2010: figs 211, A–E).	***Myrmica ritae* Emery**
−	Seen at magnification × 100, surface of head weakly micropunctate. Pronotum coarsely reticulate. Head brownish red, not strongly contrasting with mesosoma (Radchenko and Elmes 2010: figs 189, A–E)	***Myrmica pararitae* Radchenko & Elmes**
41	Posterior dorsal surface of the head (from above in the level of the eyes) with coarse reticulations	**42**
−	Posterior dorsal surface of the head with sinuous rugae; coarse reticulations, if present, then also restricted to the posterior part	**43**
42	Frons between frontal carinae level with the eyes with only 4 coarse rugae. Petiolar node, postpetiole, and sides of mesosoma with coarse reticulation (Radchenko and Elmes 2010: figs 257, A–E)	***Myrmica sinensis* Radchenko, Zhou & Elmes**
−	Frons between frontal carinae level with the eyes with ≥ 6 coarse rugae. Petiolar node, postpetiole and sides of mesosoma with coarse rugae (Radchenko and Elmes 2010: figs 301, A–E)	***Myrmica urbanii* Radchenko & Elmes**
43	Frons between frontal carinae level with the eyes with 4 very coarse rugae (Radchenko and Elmes 2010: figs 202, A–E)	***Myrmica pulchella* Santschi**
−	Frons between frontal carinae level with the eyes with ≥ 6 fine rugae (Radchenko and Elmes 2010: figs 251, A–E)	***Myrmica serica* Wheeler**
44	Petiole and postpetiole with fine sculptures, distinctly contrasting with much coarser sculptures on the mesosoma	**45**
−	Petiole and postpetiole with coarser sculptures, similar to those on the mesosoma	46
45	Frontal carina extending back to behind the eyes, posterior part of the head with reticulation; posterior and lateral margins of head with long hairs; propodeal lobes projecting apically, forming long and pointed triangles; petiole and postpetiole dull, with dense punctures (Radchenko and Elmes 2010: figs 69, A–E)	***Myrmica draco* Radchenko, Zhou & Elmes**
−	Frontal carina extending back to the posterior margin of the head, occiput without reticulation; only posterior margins of head with 0−2 long suberect hairs; propodeal lobes projecting apically, forming short and blunt triangles; petiole and postpetiole shiny, with fewer punctures (figs 26–28)	***Myrmica wangi* sp. n.**
46	Dorsum of propodeum behind the metanotal groove with a distinct U−shaped coarse rugae; first gastral tergite with clear superficial hexagonal microsculpture (figs 23–25)	***Myrmica oui* sp. n.**
−	Dorsum of propodeum behind the metanotal groove without an U−shaped coarse rugae; Gaster smooth and shiny	**47**
47	Dorsal surface of the head between rugae not punctate (Radchenko and Elmes 2010: figs 20, A–E).	***Myrmica angulata* Radchenko, Zhou & Elmes**
−	Dorsal surface of the head between rugae dull and punctate (Radchenko and Elmes 2010: figs 200, A–E)	***Myrmica poldii* Radchenko & Rigato**
48	Antennal scape distinctly angled at the base. Propodeal spines directed backward and upwards, and distinctly curved inward. Anterior surface of petiole steep, meeting the dorsal one through a sharp acute angle, dorsal plate flat, well-developed, strongly inclined backward (Radchenko and Elmes 2010: figs 22, A–E)	***Myrmica angulinodis* Ruzsky**
−	Antennal scape gradually curved at the base or at most very slightly angled. Propodeal spines not curved inward. Anterior surface of petiole meeting the dorsal one at most through a slightly rounded or obtuse angle, never sharp acute angle	**49**
49	Antennal scape always with ridge on the inner margin at the base	**50**
−	Antennal scape at most very slightly angled at the base, usually without a ridge on the inner margin	**51**
50	Sides of petiolar node with coarse rugae very similar to those on the mesosoma. Metanotal groove distinct, often deep. Petiole with short peduncle, anterior surface almost straight, meeting the dorsal one through a right or somewhat obtuse angle; dorsal plate well-developed, flattened, not inclined posteriorly (Radchenko and Elmes 2010: figs 273, A–E)	***Myrmica sulcinodis* Nylander**
−	Sides of petiolar node with punctures and short rugae less coarse than those on the mesosoma. Metanotal groove very weak or absent. Anterior surface of petiole concave, meeting the dorsal one through a rounded angle, dorsum of node somewhat convex and steeply sloping backward (figs 14–16)	***Myrmica liui* sp. n.**
51	Smaller species: HW < 1.00, AL < 1.60. Frontal carinae strongly curved at their anterior third, frontal lobes strongly extended, wide and nearly square, FLI > 1.20 (Radchenko and Elmes 2010: figs 284, A–E)	***Myrmica tibetana* Mayr**
−	Bigger species: HW > 1.15, AL > 1.90. Frontal carinae feebly curved along the whole length, frontal lobes not extended, relatively narrow, FLI < 1.15	**52**
52	Dorsum of head with very dense, but not coarse longitudinal rugae and reticulation, surface between rugae dull and densely punctate (Radchenko and Elmes 2010: figs 145, A–E)	***Myrmica kurokii* Forel**
−	Dorsum of head with not so dense rugae, never with reticulation, surface between rugae somewhat shiny and more sparsely punctuated (Radchenko and Elmes 2010: figs 143, A–E).	***Myrmica kozlovi* Ruzsky**

### Key to *pachei*-group species of *Myrmica* from the Old World

**Table:** 

1	Basal third of first gastral tergite densely punctated and longitudinally ruguloso-striated	***Myrmica sculptiventris* Radchenko & Elmes**
−	Whole surface of first gastral tergite smooth	**2**
2	Whole mesosoma dorsum with straight transversal rugae	**3**
−	Only part of mesosoma dorsum with straight transversal rugae	**5**
3	Head distinctly elongate, suboval, with narrowly rounded posterior corners; posterior third of head dorsally without longitudinal rugae and reticulation, but densely punctuate	***Myrmica yani* sp. n.**
−	Head slightly longer than broad, subsquare, with distinctly marked posterior corners; whole head dorsally with longitudinal rugae	**4**
4	Lateral and posterior margins of the head with long numerous suberect to erect hairs. Colour lighter, head dorsum dark reddish brown, mesosoma and gaster brownish red	***Myrmica villosa* Radchenko & Elmes**
−	Lateral margins of head with short decumbent hairs, long suberect hairs present only on the posterior margin and genae. Colour darker, whole body dark reddish brown	***Myrmica pachei* Forel**
5	Lateral and posterior margins of head with long numerous suberect to erect hairs	**6**
−	Lateral margins of head either glabrous or with short decumbent hairs; if long erect to suberect hairs occur, they are restricted to the posterior margin and genae	**12**
6	Head slightly longer than broad (CI < 1.15), subsquare, with distinctly marked posterior corners	**7**
−	Head distinctly elongate (CI > 1.20), suboval, with barely marked posterior corners	**9**
7	Number of rugae between frontal carinae level with the eyes >30; mesonotal and propodeal dorsum with > 35 fine transverse rugae	***Myrmica pleiorhytida* Radchenko & Elmes**
−	Number of rugae between frontal carinae level with the eyes ≤ 25; mesonotal and propodeal dorsum fine transverse rugae < 20.	**8**
8	Petiole with a stronger triangular ventral process; propleuron with rugose; mesonotal and propodeal dorsum with about 20 moderately coarse transverse sinuous rugae	***Myrmica dongi* sp. n.**
−	Petiole with a normal triangular ventral process; propleuron only with densely punctuated; mesonotal and propodeal dorsum with 8−10 coarse sinuous transverse rugae	***Myrmica mifui* sp. n.**
9	Mesonotal and propodeal dorsum with ≤20 coarser transverse rugae	**10**
−	Mesonotal and propodeal dorsum with ≥ 25 finer transverse rugae	**11**
10	Petiole low, PI1 1.68, its node with elongate flattened dorsum	***Myrmica weii* Radchenko & Zhou**
−	Petiole higher, PI1 < 1.55, its node with short, slightly convex, gradually sloping posteriorly dorsal surface	***Myrmica multiplex* Radchenko & Elmes**
11	Frontal carinae merge with the rugae that extend back to the posterior head margin. Surface of head dorsum between rugae appears shiny	***Myrmica yunnanensis* Radchenko & Elmes**
−	Frontal curved outwards to merge with the rugae that surround antennal sockets. Surface of the head dorsum between rugae appears dull, coarsely and densely punctuated	***Myrmica heterorhytida* Radchenko & Elmes**
12	Propodeal spines long (ESLI > 0.45), massive, widened at the base and often downcurved on their distal third	***Myrmica taibaiensis* Wei, Zhou & Liu**
−	Propodeal spine shorter (ESLI < 0.40), slender and straight	**13**
13	Head dorsum densely punctate, dull	**14**
−	Head dorsum not punctate, shiny	**17**
14	Head distinctly elongate (CI > 1.20), suboval, with barely marked posterior corners; dorsum of head posterior to eyes with reticulation, rest of head dorsum with longitudinal rugosity	***Myrmica polyglypta* Radchenko & Rigato**
−	Head slightly longer than broad (CI < 1.15), subsquare, with distinctly marked posterior corners; only occiput and temples with fine reticulation	**15**
15	Basal third of first gastral tergite with fine superficial hexagonal sculpture; posterior margin without any erect to suberect long hairs; dorsum of petiolar node with a distinct broad dorsal plate	***Myrmica huaii* sp. n.**
−	Whole gastral tergite smooth and shiny; posterior margin with erect to suberect long hairs; dorsum of petiolar node without a distinct broad dorsal plate	**16**
16	Lateral margins of head posterior to eyes with abundant short decumbent hairs; Mesonotal and propodeal dorsum with > 25 moderately thin (not coarse) transverse sinuous rugae; Petiole with relatively short but distinct peduncle, its anterior surface concave, dorsum of node quite narrowly rounded	***Myrmica schulzi* Radchenko & Elmes**
−	Lateral margins of head posterior to the eyes without short decumbent hairs; Mesonotal and propodeal dorsum with < 20 quite coarse transverse sinuous rugae; petiole low, with distinct peduncle, its anterior surface concave, dorsum of node widely rounded.	***Myrmica phalacra* Radchenko & Elmes**
17	Propodeal dorsum with fine longitudinal striations	***Myrmica elmesi* Bharti & Sharma**
−	Propodeal dorsum with transverse rugae.	**18**
18	Propodeal dorsum with transverse rugosity, mesonotal dorsum with short, broken irregular rugae and reticulation	***Myrmica varisculpta* Radchenko & Rigato**
−	Propodeal dorsum with sinuous longitudinal rugae, mesonotal dorsum with transverse rugosity.	***Myrmica hlavaci* Radchenko & Elmes**

## Supplementary Material

XML Treatment for Myrmica luteola

XML Treatment for Myrmica forcipata

XML Treatment for Myrmica dongi

XML Treatment for Myrmica liui

XML Treatment for Myrmica huaii

XML Treatment for Myrmica mifui

XML Treatment for Myrmica oui

XML Treatment for Myrmica wangi

XML Treatment for Myrmica yani

## References

[B1] Arnol'di KV (1934) Studien über die Systematik der Ameisen. 8. Vorläufige Ergebnisse einer biometrischen Untersuchung einiger *Myrmica*−arten aus dem europäischen Teile der USSR. Folia Zoologica et Hydrobiologica 6: 151−174.

[B2] Arnol'di KV (1976) Murav'i roda *Myrmica* Latr. srednei azii i yuzhnogo Kazakhstana [Ants of the genus *Myrmica* Latr. from Middle Asia and the southern Kazakhstan]. Zoologicheskii Zhurnal 55: 547−558. [In Russian]

[B3] Bharti H (2012a) *Myrmica nefaria* sp. n. (Hymenoptera: Formicidae) a new social parasite from Himalaya. Myrmecological News 16: 149−156. doi: 10.3897/zookeys.124.1586

[B4] Bharti H (2012b) Two new species of the genus *Myrmica* (Hymenoptera: Formicidae: Myrmicinae) from the Himalaya. Tijdschrift voor Entomologie 155: 9–14. doi: 10.1163/004074912X631742

[B5] Bharti H Sharma YP (2011a) *Myrmica elmesi* (Hymenoptera, Formicidae) a new species from Himalaya. ZooKeys 124: 51−58. doi: 10.3897/zookeys.124.1586 21998533 10.3897/zookeys.124.1586 PMC3175119

[B6] Bharti H Sharma YP (2011b) *Myrmica radchenkoi*, a new species of Ant (Hymenoptera: Formicidae) from Indian Himalaya. Sociobiology 58: 427−434.

[B7] Bharti H Sharma YP (2011c) *Myrmica longisculpta*, a new species from Himalaya (Hymenoptera: Formicidae: Myrmicinae). Acta Entomologica Musei Nationalis Pragae 51: 723−729.

[B8] Bharti H Sharma YP (2013) Three new species of genus *Myrmica* from Himalaya. Journal of Asia−Pacific Entomology 16: 123−130. doi: 10.1016/j.aspen.2012.12.006

[B9] Bolton B (1995) A new general catalogue of the ants of the world. Cambridge, Mass., Harvard University Press, 504 pp.

[B10] Bolton B (1988) A new socially parasitic *Myrmica*, with a reassessment of the genus. Systematic Entomology 13: 1−11. doi: 10.1111/j.1365-3113.1988.tb00223.x

[B11] Bolton B (2014) Catalogue of Ants of the World. 1 JULY 2014. Downloaded from http://www.antwiki.org/wiki/Species_Accounts [on 30 April 2015]

[B12] Chang YD He DH (2001a) A taxonomic study of the ant genus *Myrmica* Latreille (Hymenoptera: Formicidae: Myrmicinae) in northwestern regions of China. Journal of Ningxia Agricultural College 22: 1–9.

[B13] Chang YD He HD (2001b) Three new record species of the ant genus *Myrmica* (Hymenoptera: Formicidae). Acta Zootaxonomica Sinica 26(3): .

[B14] Chen ZP Zhou SY (2007) Molecular systematic study on twelve species of seven genera in Myrmicinae (Hymenoptera: Formicidae) from Guangxi, South China. Acta Entomologica Sinica 50: 395–404.

[B15] Chen YW (2008) Preliminary list of Formicidae in Gansu Province. Journal of Anhui Agricultural Sciences 36: 14133–14134.

[B16] Chou LY Terayama M (1991) Name lists of insects in Taiwan − Hymenoptera: Apocrita: Formicidae. Chinese Journal of Entomology 11(1): 75−84.

[B17] Collingwood C (1962) Some ants (Hym. Formicidae) from North−East Asia. Entomologisk Tidskrift 83: 215–230.

[B18] Collingwood C (1979) The Formicidae of Fennoscandia and Denmark. Fauna Entomologica Scandinavica 8: 1−174.

[B19] Collingwood C Heatwole H (2002) Ants from northwestern China (Hymenoptera, Formicidae). Psyche (Cambridge) 103: 1−24. doi: 10.1155/2000/97127

[B20] Curtis J (1854) On the genus *Myrmica* and other indigenous ants. Transactions of the Linnean Society of London 21: 211−220. doi: 10.1111/j.1096-3642.1852.tb00456.x

[B21] Czekes Z Radchenko AG Csősz S SzászLen A Tăuşan I Benedek K Markó B (2013) The genus *Myrmica* Latreille, 1804 (Hymenoptera: Formicidae) in Romania: distribution of species and key for their identification. Entomologica Romanica 17: 29−50.

[B22] Donisthorpe H (1929) The Formicidae taken by Major RGW, Hingston MC, I.M.S. (ret.), on the Mount Everest Expedition, 1924. Annals and Magazine of Natural History 4: 444−449. doi: 10.1080/00222932908673079

[B23] Eidmann H (1941) Zur Ökologie und Zoogeographie der Ameisenfauna von Westchina und Tibet. Wissenschaftliche Ergebnisse der 2. Brooke Dolan−Expedition, 1934−1935. Zeitschrift für Morphologie und Ökologie die Tiere 38: 1−43. doi: 10.1007/bf02176174

[B24] Elmes GW Radchenko AG (1998) Ants of the genus *Myrmica* from Taiwan (Hymenoptera: Formicidae). Chinese Journal of Entomology 18: 217−224.

[B25] Elmes GW Radchenko AG (2009) Two new Himalayan ant species related to *Myrmica indica*. Vestnik Zoologii 43: 107−119. doi: 10.2478/v10058-009-0006-x

[B26] Elmes GW Radchenko AG Aktaç N (2002) Four new *Myrmica* species (Hymenoptera: Formicidae) from Turkey. Annales Zoologici (Warsaw) 52: 157−171.

[B27] Elmes GW Radchenko AG Kim BJ (2001) Two new species of *Myrmica* (Hymenoptera, Formicidae) from Korea. Korean Journal of Biological Sciences 5: 107−112. doi: 10.1080/12265071.2001.9647590

[B28] Finzi B (1926) Le forme europee del genere *Myrmica* Latr. Primo contributo. Bolletino della Società Adriatica di Scienze Naturali 29: 71−119.

[B29] Francoeur A (1981) Le groupe néarctique *Myrmica lampra*. Canadian Entomologist 113: 755−759. doi: 10.4039/Ent113755-8

[B30] Francoeur A (2007) The *Myrmica punctiventris* and *M. crassirugis* species groups in the Nearctic region. In: Snelling RR Fisher BL Ward PS (Eds) Advances in ant systematics: homage to E.O. Wilson – 50 years of contributions. Memoirs of the American Entomological Institute 80: 153−185.

[B31] Guénard B Dunn RR (2012) A checklist of the ants of China. Zootaxa 3358: 1−77.

[B32] Huang JH Chen B Zhou SY (2005) A preliminary list of the family Formicidae (Insecta: Hymenoptera) from Hunan province, China. In: Guodong Ren Runzhi Zhang Fuming Shi (Eds) Classification and diversity of insects in China. China Agriculture Science and Technology Press, 394–398.

[B33] Huang RX Ouyang T Wu W Fan ZT (2004) Forty Two New Record Species of Family Formicidae (Hymenoptera: Formicoidea) from Xinjiang, China. Entomotaxonomia 26(2): 156−160.

[B34] Huang JH Zhou SY (2007) A Check list of Family Formicidae of China – Myrmicinae (Part II) (Insecta: Hymenoptera). Journal of Guangxi Normal University, Natural Science Edition 25(1): 91−99.

[B35] Kupyanskaya AN (1986a) Murav'i gruppy *Myrmica* lobicornis na dal'nem Vostoke [The ants (Hymenoptera, Formicidae) of the *Myrmica lobicornis* - group of the Far East]. In: Ler PA Kupyanskaya AN Sistematika i ekologia nasekomyh Dal'nego Vostoka, Vladivostok, DVO AN SSSR, 83−90.

[B36] Kupyanskaya AN (1986b) Murav'i severnoi chasti dal'nego Vostoka [The ants (Hymenoptera, Formicidae) of the northern part of the Far East.]. In: Ler PA Kupyanskaya AN Sistematika i ekologia nasekomyh Dal'nego Vostoka, Vladivostok, DVOAN SSSR, 91−102.

[B37] Kupyanskaya AN (1990) Murav'i Dal'nego Vostoka SSR [The ants of the Far East.]. Vladivostok, DVO AN SSSR, 258 pp.

[B38] Kutter H (1970) Über den Formenreichtum bei *Myrmica lobicornis* − Arbeiterinnen (Hymenoptera, Formicidae). Mitteilungen der Schweizerischen Entomologischen Gesellschaft 43(2): 143−146.

[B39] Kutter H (1973) Über die morphologischen Beziehungen der Gattung *Myrmica* zu ihren Satellitengenera Sifolinia Em., Symbiomyrma Arnoldi und Sommimyrma Menozzi. Mitteilungen der Schweizerischen Entomologischen Gesellschaft 46: 253−268.

[B40] Liu M Wei J Wei Z He H (1999) Studies of ant fauna in Shaanxi Province. Journal of Northwest Forestry University 14: 39–44.

[B41] Li SP Liu FL Kang J Wang YL (2005) Hymenoptera Formicidae insect name record in Henan Province. Journal of Henan Agricultural Sciences 5: 33–36.

[B42] Lyu DP Cho WS (2003) Review of Korean Formicoxenini (Hymenoptera: Formicidae: Myrmicinae) in Korea. Insecta Koreana 20: 265–280.

[B43] Ma LB Xu SQ (2011) A new ant species of the genus *Myrmica* from China (Hymenoptera: Formicoidea). Acta Zootaxonomica Sinica 36(3): 795−798.

[B44] Ma Y Xin M Song L He D (2008) A survey of ants (Hymenoptera: Formicidae) species and distribution in Ningxia. Journal of Agricultural Sciences 29: 35−38.

[B45] Mayr G (1889) Insecta in itinare Cl. Przewalski in Asia centrali novissime lecta. 17. Formiciden aus Tibet. Trudy Russkago Entomologicheskago Obshchestva 24: 278−280.

[B46] Nylander W (1846a) Adnotationes in monographiam formicarum borealium Europae. Acta Societatis Scientiarum Fennicae 2: 875−944.

[B47] Nylander W (1846b) Additamentum adnotationum in monographiam formicarum borealium Europae. Acta Societatis Scientiarum Fennicae 2: 1041−1062.

[B48] Nylander W (1849) Additamentum alterum adnotationum in monographiam formicarum borealium. Acta Societatis Scientiarum Fennicae 3: 25−48.

[B49] Nylander W (1856) Synopsis des formicides de France et d'Algérie. Annales des Sciences Naturelles (Zoologie) 5(4): 51−109.

[B50] Nylander W (1857) Untitled note introduced by “M. L. Fairmaire communique la note suivante de M. Nylander sur les Formicides du Mont−Dore, et la Société en décide l'impression dans le Bulletin". Annals de la Société Entomologique de France, Bulletins Trimestriels (3) 4(1856): 78−79.

[B51] Radchenko AG (1994a) New Palaearctic species of the genus *Myrmica* Latr. (Hymenoptera, Formicidae). Memorabilia Zoologica 48(1): 207−217.

[B52] Radchenko AG (1994b) Taksonomicheskaya struktura roda *Myrmica evrazii*. Soobshchenie 1. Zoologichesky Zhurnal 73(6): 39−51. [In Russian; for English translation see Radchenko AG 1995a. Taxonomic structure of the ant genus *Myrmica* (Hymenoptera, Formicidae) of Eurasia. Comunication 1. Entomological Review (Washington) 74(3): 91−106.]

[B53] Radchenko AG (1994c) Opredelitel'naya tablitsa murav'ev roda *Myrmica* tsentral'noi i vostochnoi palearktiki. Zoologicheskii Zhurnal 73(7−8): 130−145. [In Russian; for English translation see Radchenko AG 1995b: A key to the species of the genus *Myrmica* (Hymenoptera, Formicidae) of the Central and Eastern Palaearctic region. Entomological Review (Washington) 74 (3): 154−169.]

[B54] Radchenko AG (1994d) Obzor vidov gruppy scabrinodis roda *Myrmica* tsentral'noi i vostochnoi palearktiki. Zoologicheskii Zhurnal 73(9): 75−82. [In Russian; for English translation see Radchenko AG 1995c: A review of species in the *scabrinodis*−group of the genus *Myrmica* Latreille (Hymenoptera, Formicidae) of the Central and Eastern Palaearctic. Entomological Review (Washington) 74 (5): 116−124.]

[B55] Radchenko AG (1994e) Obzor vidov grupp rubra, rugosa, arnoldii, luteola i schencki roda *Myrmica* tsentral'noi i vostochnoi palearktiki. Zoologicheskii Zhurnal 73(11): 72−80. [In Russian; for English translation see Radchenko AG 1995d: A survey of species of *Myrmica* of groups of *rubra*, *rugosa*, *arnoldii*, *luteola* and *schencki* (Hymenoptera, Formicidae) from Central and Eastern Palaearctic. Entomological Review (Washington) 74 (8): 122−132.]

[B56] Radchenko AG (1994f) Obzor vidov gruppy lobicornis roda *Myrmica* tsentral'noi i vostochnoi palearktiki. Zoologicheskii Zhurnal 73(11): 81−92. [In Russian; for English translation see Radchenko AG 1996. A review of species of *Myrmica* belonging to the group of *lobicornis* (Hymenoptera, Formicidae) from the Central and Eastern Palaearctic. Entomological Review (Washington) 74 (8): 133−146.]

[B57] Radchenko AG Czechowski W Czechowska W (1997) The genus *Myrmica* Latr. in Poland – a survey of species and a key for their identification. Annales Zoologici (Warsaw) 47: 481−500.

[B58] Radchenko AG Elmes GW (1998) Taxonomic revision of the *ritae* species−group of the genus *Myrmica* (Hymenoptera, Formicidae). Vestnik Zoologii 32(4): 3−27.

[B59] Radchenko AG Elmes GW (1999a) Ten new species of *Myrmica* from the Himalaya. Vestnik Zoologii 33(3): 27−46.

[B60] Radchenko AG Elmes GW (1999b) First description of the female of *Myrmica ritae*, with some notes on the *ritae*−group. Vestnik Zoologii 33(6): 95−98.

[B61] Radchenko AG Elmes GW (2001a) First record of the genus *Myrmica* from northern Vietnam, with a description of two new species. Annales Zoologici (Warsaw) 51: 221−225.

[B62] Radchenko AG Elmes GW (2002) First descriptions of the sexual forms of seven Himalayan *Myrmica* species. Vestnik Zoologii 36(5): 35−46.

[B63] Radchenko AG Elmes GW (2003b) *Myrmica afghanica*, a new ant species from Afghanistan. Zootaxa 375: 1−8.

[B64] Radchenko AG Elmes GW (2009a) Taxonomic revision of the *pachei* species−group of the genus *Myrmica* Latreille (Hymenoptera: Formicidae). Annales Zoologici (Warsaw) 59: 67−92. doi: 10.3161/000345409X432592

[B65] Radchenko AG Elmes GW (2009b) Important alterations in the taxonomy of the ant genus *Myrmica*, based on the investigation of the M. Ruzsky's type specimens, preserved in the Museo Civico di Storia Naturale “Giacomo Doria" in Genoa. Annali del Museo Civico di Storia Naturale “Giacomo Doria" 100: 501−525.

[B66] Radchenko AG Elmes GW (2009c) *Myrmica emeryi*, a new ant species from South−East Asia. Doriana 8(361): 1−7.

[B67] Radchenko AG Elmes GW (2010) *Myrmica* ants (Hymenoptera, Formicidae) of the Old World. Fauna Mundi 3: 1−789.

[B68] Radchenko AG Elmes GW Savolainen R (2008a) *Myrmica xavieri* sp. n., a new ant species from Spain. Entomologica Fennica 19: 49−54.

[B69] Radchenko AG Elmes GW Viet BT (2006) Ants of the genus *Myrmica* (Hymenoptera: Formicidae) from Vietnam, with a description of a new species. Myrmecologische Nachrichten 8: 35−44.

[B70] Radchenko AG Elmes GW Woyciechowski M (2002) An appraisal of *Myrmica bergi* Ruzsky, 1902 and related species. Annales Zoologici (Warszawa) 52: 409−421.

[B71] Radchenko AG Elmes GW Zhou SY Elmes GW (2001b) New and rare *Myrmica* species (Hymenoptera: Formicidae) from southern China. Annales Zoologici (Warsaw) 51: 211−219.

[B72] Radchenko AG Zhou SY Elmes GW Rigato F (2008b) Seven new *Myrmica* species (Hymenoptera: Formicidae) from China. Annales Zoologici (Warsaw) 58: 767−784. doi: 10.3161/000345408X396701

[B73] Ruzsky M (1915) O murav'yakh Tibeti i yuzhnoi Gobi. Po materialam sobrannym ekspeditsiei polkovnika P.K. Kozlova. Ezhegodnik Zoologicheskago Muzeya Imperatorskoi Akademii Nauk 20: 418−444.

[B74] Sadil JV (1952) A revision of the Czechoslovak forms of the genus *Myrmica* Latreille. Sborník Entomologického Oddelení Národního Musea v Praze 27: 233−278.

[B75] Santschi F (1931) Notes sur le genre *Myrmica* Latreille. Revue Suisse de Zoologie 38: 335−355.

[B76] Santschi F (1937) Fourmis du Japon et de Formose. Bulletin et Annales de la Société Entomologique de Belgique 77: 361−388.

[B77] Stappen MV (2014) *Myrmica* ants (Hymenoptera: Formicidae) of the Old World (review). Asian Myrmecology 6: 129−132.

[B78] Seifert B (1988) A taxonomic revision of the *Myrmica* species of Europe, Asia Minor, and Caucasus. Abhandlungen und Berichte des Naturkundemuseums Görlitz 62(3): 1−75.

[B79] Seifert B (2003) The Palaearctic members of the *Myrmica schencki* group with description of a new species. Beiträge zur Entomologie 53: 141−159.

[B80] Seifert B (2011) A taxonomic revision of the Eurasian *Myrmica salina* species complex. Soil Organisms 83(2): 169−186.

[B81] Tang J Li S Huang EY Zhang BY Chen Y (1995) Economic insect fauna of China. Fascicule 47. Hymenoptera: Formicidae (1). Science Press, Beijing, 134 pp.

[B82] Tie R Xu S (2004) Variety and distribution of ants in Northwest China. Journal of Ningxia Agricultural College 25: 4–9.

[B83] Tie R Xu S (2005) Ant species list in central part of Mt Qinling. Sichuan Journal of Zoology 24: 46.

[B84] Viehmeyer H (1922) Neue Ameisen. Archiv für Naturgeschichte 88(A.7): 203−220.

[B85] Wang W (2007) Fauna of Formicidae ants from three nature reserves in southwest Hubei. Chinese Bulletin of Entomology 44: 267–270.

[B86] Wang W Shen ZK Zhao YH (2009) A taxonomic study on the family Formicidae from Hubei Province (Insecta: Hymenoptera: Formicidae). China University of Geosciences Press CO., LTD, Wuhan, 210 pp.

[B87] Wang W Zhou SY Huang JH (2005) A new species of the genus *Vollenhovia* Mayr and a new record species of the genus *Myrmica* Latreille from China (Hymenoptera, Formicidae). Acta Zootaxonomica Sinica 30: 835−838. [In Chinese]

[B88] Weber NA (1947) A revision of the North American ants of the genus *Myrmica* Latreille with a synopsis of the Palaearctic species. 1. Annals of the Entomological Society of America 40: 437–474. doi: 10.1093/aesa/40.3.437

[B89] Weber NA (1948) A revision of the North American ants of the genus *Myrmica* Latreille, with a synopsis of the Palearctic species. 2. Annals of the Entomological Society of America 41: 267−308. doi: 10.1093/aesa/41.2.267

[B90] Weber NA (1950) A revision of the North American ants of the genus *Myrmica* Latreille with a synopsis of the Palearctic species. 3. Annals of the Entomological Society of America 43: 189−226. doi: 10.1093/aesa/43.2.189

[B91] Wei C Zhou SY Liu MT (1999) A new record species of the genus *Myrmica* Latreille (Hymenoptera: Formicidae) from Shaanxi, China. Entomotaxonomia 21: 60.

[B92] Wei C He H Liu M (2001a) A study on ants species composition and ant fauna of Mt. Taibai. Scientia Silvae Sinicae 37: 129–134.

[B93] Wei C Zhou SY He H Liu MT (2001b) A taxonomic study of the genus *Myrmica* Latreille from China. (Hymenoptera: Formicidae). Acta Zootaxonomica Sinica 26: 560−564.

[B94] Wheeler WM (1928) Ants collected by Professor F. Silvestri in China. Bollettino del Laboratorio di Zoologia Generale e Agraria della Reale Scuola Superiore d'Agricoltura. Portici 22: 3−38.

[B95] Wheeler WM (1929) Ants collected by Professor F. Silvestri in Formosa, the Malay Peninsula and the Philippines. Bollettino del Laboratorio di Zoologia generale e agraria del R. Istituto superiore agrario di Portici 24: 27−64.

[B96] Wheeler WM (1930a) Formosan ants collected by Dr. R. Takahashi. Proceedings of the New England Zoological Club 11: 93−106.

[B97] Wheeler MW (1930b) A list of the known Chinese ants. Peking Natural History Bulletin 5: 53−81.

[B98] Wu CF (1941) Superfamily Formicoidea – Family Formicidae. Catalogus Insectorum Sinensium 6: 141−204.

[B99] Wu W Li XM Guo H (2004) A primary study on the fauna of Formicidae in Urumqi and its vicinities. Arid Zone Research 21: 179–182.

[B100] Wu J Wang C (1995a) The ants of China. China Forestry Publishing House, Beijing, 214 pp [In Chinese]

[B101] Xia Y Zheng Z (1997a) A survey of Formicidae from Xinjiang. Journal of Shaanxi Normal University. Natural Science Edition 25(2): 64−66. [In Chinese]

[B102] Xin M Ma Y He D (2011) Fauna composition of Formicidae in Ningxia. Journal of Ningxia University (Natural Science Edition) 32: 403−412.

[B103] Xu ZH (2002) A study on the biodiversity of Formicidae ants of Xishuangbanna Nature Reserve. Yunnan Science and Technology Press, Kunming, 181 pp.

[B104] Xu ZH Chu JJ Zhang CL Yu NN (2011) Ant species and distribution pattern in Gongbo Nature Reserve in Southeastern Tibet. Sichuan Journal of Zoology 30: 118−123.

[B105] Yarrow IHH (1955) The type species of the ant genus *Myrmica* Latreille. Proceedings of the Royal Entomological Society of London (B) 24: 113−115. doi: 10.1111/j.1365-3113.1955.tb01486.x

[B106] Yasumatsu K (1941) Ants collected by Mr. H. Takahasi in Hingan (Hsingan) North Province, North Manchuria (Hymenoptera, Formicidae). Journal of the Natural History Society of Taiwan 31: 182−185.

[B107] Zhang C Xu Z Yang B Chu J Yu N (2011) Species composition and diversity of ant communities in Mount Sejila in Southeastern Xizang (Tibet). Journal of Northeast Forestry University 39: 79−82.

[B108] Zhou SY (2001) Ants of Guangxi. Guangxi Normal University Press, Guilin, China, 255 pp [In Chinese]

[B109] Zhou SY (2005) Hymenoptera: Formicidae. In: Yang MF Jin DC (Eds) Insects from Dashahe Nature Reserve of Guizhou. Guizhou Peoples Publishing House, Guiyang, 480−482.

[B110] Zhou SY (2006) Formicidae. In: Li ZZ Jin DC (Eds) Insects from Fanjingshan Landscape. Guizhou Science and Technology Publishing House, Guiyang, 579−589.

[B111] Zhou SY (2013) Formicidae. In: Li ZZ Jin DC (Eds) Insects from Leigongshan Landscape. Guizhou Science and Technology Publishing House, Guiyang, 634−646.

[B112] Zhou SY Huang JH (2002) Hymenoptera: Formicidae. In: Shen XC Zhao YQ (Eds) The fauna & taxonomy of insects in Henan. Volume 5. Insects of the Mountains Taihang and Tongbai regions. China Agricultural Science & Technology Press, Beijing, 428−431.

[B113] Zhou SY Qian F (2010) Hymenoptera: Formicidae. In: Chen XS Li ZZ Jin DC (Eds) Insects from Mayanghe Landscape. Guizhou Science & Technology Publishing House, Guiyang, 426−441.

